# Distinctive binding properties of human monoclonal LGI1 autoantibodies determine pathogenic mechanisms

**DOI:** 10.1093/brain/awaa104

**Published:** 2020-05-21

**Authors:** Melanie Ramberger, Antonio Berretta, Jeanne M M Tan, Bo Sun, Sophia Michael, Tianrong Yeo, Jakob Theorell, Rachael Bashford-Rogers, Sofija Paneva, Victoria O’Dowd, Neesha Dedi, Sarfaraj Topia, Robert Griffin, Jorge Ramirez-Franco, Oussama El Far, Stéphanie Baulac, Maria I Leite, Arjune Sen, Alexander Jeans, David McMillan, Diane Marshall, Daniel Anthony, Daniel Lightwood, Patrick Waters, Sarosh R Irani

**Affiliations:** a1 Oxford Autoimmune Neurology Group, Nuffield Department of Clinical Neurosciences, University of Oxford, Oxford, UK; a2 Department of Neurology, John Radcliffe Hospital, Oxford University Hospitals, Oxford, UK; a3 Department of Neurology, National Neuroscience Institute, 11 Jalan Tan Tock Seng, Singapore 308433, Singapore; a4 Experimental Neuropathology Group, Department of Pharmacology, University of Oxford, Oxford, UK; a5 Wellcome Centre for Human Genetics, University of Oxford, Oxford, UK; a6 UCB Pharma, 208-216 Bath Road, Berkshire, UK; a7 Unité de Neurobiologie des Canaux Ioniques et de la Synapse, INSERM UMR_S 1072, Aix Marseille Université, Marseille, France; a8 Sorbonne Université, UPMC Univ Paris 06, UMR S 1127, INSERM, U1127, CNRS, UMR 7225, Institut du Cerveau et de la Moelle épinière (ICM), Hôpital Pitié-Salpêtrière, Paris, France; a9 Oxford Epilepsy Research Group, University of Oxford, Oxford, UK

**Keywords:** LGI1, encephalitis, autoimmune, antibody, immunology

## Abstract

Autoantibodies against leucine-rich glioma inactivated 1 (LGI1) are found in patients with limbic encephalitis and focal seizures. Here, we generate patient-derived monoclonal antibodies (mAbs) against LGI1. We explore their sequences and binding characteristics, plus their pathogenic potential using transfected HEK293T cells, rodent neuronal preparations, and behavioural and electrophysiological assessments *in vivo* after mAb injections into the rodent hippocampus. In live cell-based assays, LGI1 epitope recognition was examined with patient sera (*n = *31), CSFs (*n = *11), longitudinal serum samples (*n = *15), and using mAbs (*n = *14) generated from peripheral B cells of two patients. All sera and 9/11 CSFs bound both the leucine-rich repeat (LRR) and the epitempin repeat (EPTP) domains of LGI1, with stable ratios of LRR:EPTP antibody levels over time. By contrast, the mAbs derived from both patients recognized either the LRR or EPTP domain. mAbs against both domain specificities showed varied binding strengths, and marked genetic heterogeneity, with high mutation frequencies. LRR-specific mAbs recognized LGI1 docked to its interaction partners, ADAM22 and ADAM23, bound to rodent brain sections, and induced internalization of the LGI1-ADAM22/23 complex in both HEK293T cells and live hippocampal neurons. By contrast, few EPTP-specific mAbs bound to rodent brain sections or ADAM22/23-docked LGI1, but all inhibited the docking of LGI1 to ADAM22/23. After intrahippocampal injection, and by contrast to the LRR-directed mAbs, the EPTP-directed mAbs showed far less avid binding to brain tissue and were consistently detected in the serum. Post-injection, both domain-specific mAbs abrogated long-term potentiation induction, and LRR-directed antibodies with higher binding strengths induced memory impairment. Taken together, two largely dichotomous populations of LGI1 mAbs with distinct domain binding characteristics exist in the affinity matured peripheral autoantigen-specific memory pools of individuals, both of which have pathogenic potential. In human autoantibody-mediated diseases, the detailed characterization of patient mAbs provides a valuable method to dissect the molecular mechanisms within polyclonal populations.

## Teaser

Ramberger *et al*. generate patient-derived monoclonal antibodies against LGI1 and describe their epitopes and sequences, plus their pathogenic potential *in vitro* and *in vivo*. They find that binding characteristics of these antibodies inform the molecular mechanisms and relative pathogenicity within polyclonal specificities.

**See Zekeridou and Pittock (doi:10.1093/brain/awaa153) for a scientific commentary on this article.**

## Introduction

Autoantibodies against leucine-rich glioma inactivated 1 (LGI1) are commonly found in older male patients with limbic encephalitis. These patients often exhibit a dense amnesia alongside focal seizures including faciobrachial dystonic seizures (FBDS). In the longer term, many have residual hippocampal atrophy with memory deficits (Irani *et al.*, 2010; Lai *et al.*, 2010; Finke *et al.*, 2017). In addition, some patients with LGI1 antibodies have isolated seizures including FBDS, Morvan’s syndrome, and a few have pain and neuromyotonia (Irani *et al.*, 2010; Finke *et al.*, 2017; Gadoth *et al.*, 2017; Thompson *et al.*, 2018). Several strands of evidence support the direct pathogenicity of LGI1 antibodies. These include their binding to native, surface-exposed LGI1 epitopes, and a clear, often dramatic, response to immunotherapies including plasma exchange (Irani *et al.*, 2010, 2011, 2013; Lai *et al.*, 2010; van Sonderen *et al.*, 2016; Thompson *et al.*, 2018). Also, passive transfer of unfractionated patient serum IgGs (immunoglobulin G) can induce amnesia in rodents (Petit-Pedrol *et al.*, 2018).

LGI1 antibodies are predominantly of the IgG4 subclass (Thompson *et al.*, 2018) and are most consistently detected in patient sera, with CSF positivity found in around 80–90% of patients (van Sonderen *et al.*, 2016; Lehmann-Horn *et al*., 2020). Further, an almost universal association with HLA-DRB1*07:01 suggests a critical molecular interaction for the peripheral generation of affinity matured immunoglobulins (Kim *et al.*, 2017; van Sonderen *et al.*, 2017; Binks *et al.*, 2018; Mueller *et al.*, 2018). From a neuroscience perspective, LGI1 is reported to be a secreted neuronal protein that stabilizes the trans-synaptic complex formed between the pre- and postsynaptic receptors: ADAM23 and ADAM22, respectively (Fukata *et al.*, 2006; Ohkawa *et al.*, 2013). Established molecular events after the binding of polyclonal serum IgGs include disruption of LGI1’s interactions with ADAM22/23 and the downregulation of presynaptic K_v_1.1 channels and postsynaptic AMPA receptors (Ohkawa *et al.*, 2013; Petit-Pedrol *et al.*, 2018).

However, studies do not address the relative characteristics of mechanisms by which individual LGI1-specific antibodies, from within the polyclonal serum pool, carry pathogenic potential. Here, we isolate antibodies from patients and ask whether the binding characteristics of peripheral patient-derived LGI1 monoclonal antibodies (mAbs) may be used to dissect and reveal distinct *in vitro* and *in vivo* properties of the LGI1 specificities.

## Materials and methods

### Monoclonal antibody generation and characterization

LGI1-specific mAbs were isolated after *in vitro* activation of B cells from two patients (Tickle *et al.*, 2015). Briefly, unfractionated peripheral blood mononuclear cells (PBMCs) were differentiated into antibody-secreting cells, and LGI1-specific binding from B-cell supernatants was confirmed to both full-length LGI1 expressing HEK293T cells (Irani *et al.*, 2010) and to soluble LGI1-rabbit Fc fusion protein (sLGI1-Fc). Subsequently, cultured antibody-secreting cells were incubated with sLGI1-Fc captured onto streptavidin beads via a biotinylated goat anti-rabbit Fc reagent. After addition of fluorescently-labelled anti-human IgG Fc antibodies, LGI1-specific antibody-secreting cells were identified by the presence of a fluorescent halo, formed by the surrounding complex of locally secreted LGI1-specific antibodies. In total, antibody-secreting cells from 14 LGI1-specific wells were isolated and genes were cloned and expressed as recombinant human IgG4 antibodies (Clargo *et al.*, 2014; Tickle *et al.*, 2015).

Immunoglobulin heavy (IGH) chain gene segments [variable (V), diversity (D) and joining (J)] and immunoglobulin light chain gene (V and J) sequences were analysed using IMGT/V-QUEST (http://www.imgt.org/IMGTindex/V-QUEST.php).

Relative mAb binding characteristics were assessed using live HEK293T cells expressing full-length LGI1 with fluorescence intensity measured on a FLUOstar^®^ Omega Microplate Reader (BMG Labtech). The K_d_ values were calculated by fitting specific binding data to a one-site hyperbola non-linear regression analysis using Equation 1:

(1) $$y=\frac{B_{\max}\times [x]}{\left[x\right]+K_{d}}$$

For cross-competition assays, cells were sequentially exposed to an excess amount of the first mAb (10 μg/ml), followed by a fluorescently-labelled second mAb at 2.5 μg/ml. Preincubation with a control mAb (isotype-matched human mAb targeting A33 (Acc. No. NP_005805.1), a protein expressed in colon cancer and not in brain) was defined as 0% blocking, and preincubation with the identical LGI1 mAb was defined as 100% blocking.

### Patients and LGI1-antibody detection

**Figure 1:**
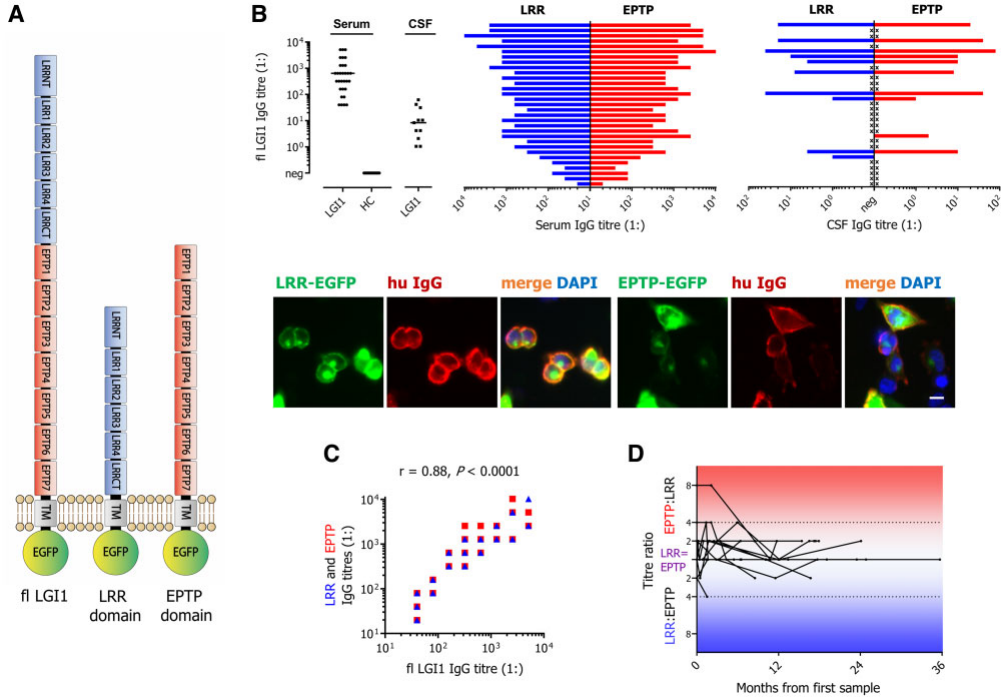
**Patient serum and CSF IgG binding to full-length LGI1, and to its LRR and EPTP domains in live cell-based assays.** (**A**) Schematic representation of human full-length (fl) LGI1, plus LRR and EPTP domain constructs. All have an N-terminal signal peptide (SP) and C-terminal transmembrane domain (TM) plus intracellular EGFP. (**B**) Sera from 31 patients (20 male; age range onset 46–86, median 63 years), plus 11 CSFs, were analysed alongside 31 age-matched healthy controls (HC). While all patient sera bound both domains of LGI1, typically at similar levels, two CSF samples bound only to LRR (blue) or EPTP (red) domains. Unavailable CSF samples are indicated by an ‘x’. Fluorescent images show a live cell-based assay with Patient 1’s serum IgG binding (hu IgG, red) to both the (EGFP-tagged) LRR and EPTP domains of LGI1. DAPI was used as a nuclear marker. Scale bar = 10 μm. (**C**) Overall, serum end-point titres to full-length LGI1 correlated strongly with the LRR- and with EPTP domain-specific antibody levels (Spearman’s *r *=* *0.88, *P < *0.0001). (**D**) Ratios of serum LRR- and EPTP-antibody end-point titres over time are shown in 15 patients, treated with corticosteroids (*n *=* *14), intravenous immunoglobulins (*n = *8), plasma exchange (*n = *5), mycophenolate mofetil (*n = *3), azathioprine (*n = *3), rituximab (*n = *1), methotrexate (*n = *1), cyclophosphamide (*n = *1), thymectomy (*n = *1) or no therapy (*n = *1). The majority of patients had a constant relative ratio of LRR:EPTP antibody titres. The main outlier had an 8-fold higher EPTP serum end-point titre, which decreased after immunotherapy and cessation of seizures.

LGI1-antibody epitopes were studied using live cell-based assays from 31 clinically characterized patients with serum LGI1 antibodies (≥⩾1:40 end point dilutions), including 11 paired CSF samples (dilutions from neat). Based on previous cloning strategies (Irani *et al.*, 2010), the coding sequence (CDS) of human full-length LGI1 (CCDS7431.1; NP_005088.1), its leucine rich repeat (LRR; residues 35–223) and its epitempin repeat (EPTP; residues 224–557) domains were independently cloned into the mammalian expression vector pcDNA3.1. All three constructs included the N-terminal signal peptide (residues 1–37), and, at the C-terminal end, the transmembrane/intracellular domains of human contactin-associated protein-like 2 (CASPR2; residues 1248–1331; to achieve surface tethering), and finally intracellular EGFP (Fig. 1A).

For staining, live transfected HEK293T cells were incubated with mAbs for 1 h at room temperature. For some experiments, soluble human full-length LGI1 (sLGI1) was expressed in HEK293T cell medium and applied to HEK293T cells expressing human ADAM22 (CCDS43609.1; NP_057435.2) or human ADAM23 (CCDS2369.1; NP_003803.1). Rat embryonic hippocampal neurons were cultured for 28–42 days *in vitro*, incubated with mAbs for 30 min at 37°C and fluorescence-conjugated detection antibodies (see below) were applied after fixation in 4% formaldehyde (plus 4% sucrose for neurons). Cell surface binding was visualized by fluorescence microscopy (Leica DM2500, Zeiss LSM710) and quantified by blindly determined end-point titres or by flow cytometry. For sections, non-perfused brains from adult male Wistar rats were fixed with 4% paraformaldehyde and stained with monoclonal antibodies (≥5 μg/ml); previous descriptions were modified using 1% hydrogen peroxide in methanol for blocking endogenous peroxidases (Irani *et al.*, 2010; Lai *et al.*, 2010).

LGI1-knockout tissue was obtained from postnatal Day 14 *Lgi1^−^^/^*^−^ mice (Chabrol *et al.*, 2010; Seagar *et al.*, 2017). Preadsorption with full-length LGI1 was used to confirm mAb specificity. For the latter, mAbs at a concentration of 200 ng/ml were incubated with either HEK293T cells expressing membrane-tethered full-length LGI1 or untransfected HEK293T sequentially for 9 × 30-min cycles.

### IgG purification

Polyclonal total serum IgG and IgG4 subfractions were purified from plasma of four LGI1 antibody-positive patients and two age-matched healthy controls [using Protein G Sepharose^®^ 4 Fast Flow, 17-0618-01; and GE Healthcare and CaptureSelect™ IgG4 (Hu) Affinity Matrix, 290005, Thermo Fisher, respectively].

### Fab′ fragment generation

Digestion of human IgG4 mAbs was performed using immobilized FabRICATOR (A0-FR6-100, Genovis) in 10 mM PBS pH 7.4 at 37°C for 1 h and antibody fragments were separated using spin columns. Digested F(ab′)2 was purified using Fc based capture select spin columns. Purified F(ab′)2 fragments were reduced to Fab′ fragments using the mild reducing agent 2-merceptoethylamine hydrochloride (M6500-25G, Sigma) at 37°C for 90 min and adjusted to a final concentration of 50 mM. Reduced Fab′ fragments were then buffer exchanged using a G25 Sephadex^®^ in PD10 desalting column (17085101, GE Healthcare) followed by alkylation of reduced SH groups using *N*-ethylamine (E3876-25G, Sigma). Alkylated Fab′ fragments were gel filtered using a Superdex 200 Increase 10/300 GL gel filtration column (28-9909-44, GE Healthcare) to remove impurities, with 99% monomeric purity measured by analytical size exclusion chromatography.

### Studies in HEK293T cells and hippocampal neurons

LGI1-specific mAbs, control mAb, Fab′ fragments, and purified serum IgG fractions were conjugated with an endosomal-pH sensitive red-fluorescent dye (pHrodo iFL Red STP ester, P36011, Thermo Fisher), following the manufacturer’s instructions. pHrodo-conjugated antibodies were applied at 37°C to HEK293T cells co-transfected to express sLGI1 and ADAM22 or ADAM23, and to hippocampal neurons. Quantification of internalized pHrodo-labelled IgG on HEK293T cells was determined by flow cytometry (Attune_NxT, FlowJo_V10). To inhibit dynamin activity, 50 μM dynasore (14062, Cayman Chemical) was added 1 h prior to and during incubation with mAbs or Fab′ fragments.

To study residual surface-bound LGI1 mAbs over time in HEK293T cells expressing sLGI1 in combination with ADAM22 or ADAM23, individual mAbs were applied for 1 h at 4°C to allow binding without inducing internalization. Subsequently, cells were incubated over varied durations at 37°C, prior to fixation. After fixation, surface-bound human IgG was detected with an Alexa Fluor^®^ 488-conjugated goat anti-human IgG secondary antibody (H + L; A-11013, Thermo Fisher). Surface-bound IgG was quantified with flow cytometry (Attune_NxT, FlowJo_V10).

For time-lapse live cell imaging, pHrodo-conjugated mAbs were applied to neurons and images at 15-min intervals were acquired from three distinct positions per well (Zeiss 880 Axio observer spinning disc confocal microscope with CSU-X1M 5000 dual cam). Images were captured (Hamamatsu Orca Flash4.0 v2 sCMOS 6.5-micron pixels) and quantified computationally. To reduce inter-frame noise, pHrodo-fluorescence from all frames was individually normalized by scaling to the median absolute deviation of that frame and centred by the median value (which was always among the lowest values). Then, the sum of intensity above background was calculated using:

(2) $$S_{\mathrm{int}}=\sum_{x=1}^{n}x\times \left[x>k\right]$$

where *k* was defined as the 99th percentile of the first four frames (i.e. the first hour) and brackets are Iverson brackets returning 1 if *x *>* k* and 0 otherwise.

For static quantification of internalization, three fields (each 709 × 709 μm) per well were captured at random using an inverted confocal microscope (Zeiss LSM710, 20× objective). After 96 h, pHrodo-positive neurons were identified by characteristic morphology and cell counting (means of three blinded observers). Fluorescence intensity was quantified using Equation 2, where *k* was defined as the 99th percentile of the control mAb. The R package pixSum, freely available from https://github.com/jtheorell/pixSum, was used for the calculations of Equation 2 results and associated image processing.

For some experiments, live hippocampal neurons were incubated with unconjugated mAbs for up to 96 h at 37°C. Before cell permeabilization, surface-bound human IgG was visualized with an Alexa Fluor^®^ 488-conjugated goat anti-human IgG secondary antibody (H + L; A-11013, Thermo Fisher), and a tertiary antibody was used to amplify the signal [Alexa Fluor^®^ 488 donkey anti-goat IgG (H + L); A-11055, Thermo Fisher]. After permeabilization (0.1% Triton™ X-100), internalized IgG was detected with an alternatively labelled secondary antibody [Alexa Fluor^®^ 568 goat anti-human IgG (H + L); A-21090, and subsequently Alexa Fluor^®^ 568 donkey anti-goat IgG (H + L); A-11057, Thermo Fisher].

To study antibodies that may block the LGI1-ADAM22/ADAM23 interaction, supernatants from HEK293T cells transfected to express sLGI1 were preincubated with increasing concentrations of individual mAbs for 1 h at room temperature, and binding of sLGI1 to ADAM22/23 expressing HEK293T cells was detected using an LRR-specific mAb (mAb02 or mAb06). To investigate similar effects in neurons, cells were preincubated with an excess of each EPTP-specific mAb (14 μg/ml) for 30 min at 37°C. Subsequently, a pHrodo-conjugated LRR-specific mAb (mAb01 at 1 μg/ml) was applied for up to 96 h to assess internalization.

### Intrahippocampal injection of monoclonal antibodies

Male C57BL6/J mice (Charles River), over 12 weeks old (28–35 g), were housed in cages of five until 1 week before surgery, when they were housed individually. The room was maintained at a controlled temperature (21°C) and humidity (5–10%) with illumination at 12-h cycles; food and water were available *ad libitum*. All experiments were performed during the light phase, and animals were habituated to the experimental room for 1 day before beginning the tests. All procedures were conducted in accordance with standard ethical guidelines and Institutional Animal Care and Use Committee (University of Oxford). On the day of surgery, mice were anaesthetized with isoflurane, and placed in a stereotactic apparatus. A mid-sagittal incision was made to expose the cranium and two burr holes were drilled over the hippocampi to the following coordinates from the bregma: anteroposterior −1.5 mm; lateral, ±1.8 mm. A glass capillary containing the solution to be injected was lowered 1.5 mm ventral to bregma, and a 0.5 μl injection of 2 mg/ml mAb was made over a 5-min period. The incision was cleaned and closed with resolvable sutures. Overall, 103 mice were used for these studies: 73 for behavioural and brain tissue studies and 30 others for electrophysiological studies.

### Western blotting

Hippocampal enriched mouse brain tissue was homogenized in 20 mM Tris-HCl (pH 8.0), 2 mM EDTA, 320 mM sucrose and protease inhibitor (11836153001, Roche). Homogenates were spun at 20 000*g* (1 h) for synaptic fraction enrichment. Pellets were resuspended in 20 mM Tris-HCl (pH 8.0), 1 mM EDTA, 100 mM NaCl, and 1.3% Triton™ X-100 and spun at 100 000*g* for 1 h. Supernatants were then separated by SDS/PAGE and blotted on nitrocellulose membranes.

### Behavioural analysis

All behavioural tasks were performed 7 days after bilateral intracranial injection and data were analysed by either blinded researchers or using the ANY-maze analysis software. Tasks were aimed to assess memory (novel object recognition in open field), anxiety (open field) and locomotor activity (clasping ability and distance travelled), and were performed as reported elsewhere (Petit-Pedrol *et al.*, 2018).

### Acute hippocampal slice electrophysiology

Field excitatory postsynaptic potentials (fEPSPs) were recorded in 300-μm thick acute hippocampal slices prepared as described (Padamsey *et al.*, 2017). Slices were placed in an interface recording chamber perfused with oxygenated artificial CSF at 1–2 ml/min, and a bipolar stimulating electrode (FHC Inc.) was inserted in Schaffer collaterals to deliver test and conditioning stimuli. A borosilicate glass recording electrode filled with artificial CSF was positioned in stratum radiatum of CA1 and responses to 0.067 Hz stimuli were recorded for at least 10 min prior to beginning experiments to ensure stability. Field potentials were amplified using a Digitimer NeuroLog amplifier, filtered below 3 Hz and above 3 kHz and digitized with a BNC-2090A converter (National Instruments). WinWCP (University of Strathclyde) software was used for recording and Clampfit (Molecular Devices) for analysis. Long-term potentiation (LTP) was induced using a 20× theta-burst protocol comprising a block of four stimuli at 100 Hz repeated 20 times over 20 s. The magnitude of fEPSPs was determined as the gradient of the rising slope to avoid population spike contamination. Paired-pulse ratios were obtained by delivering two stimuli at an interval of 50 ms and expressed as fEPSP2/fEPSP1.

### Commercial reagents, statistics and ethical approvals

Other commercially available antibodies used were: goat anti-human IgG Fcγ (31125, Thermo Fisher), AF488 goat anti-rabbit IgG (H + L; A-11008, Thermo Fisher), mouse anti-MAP2 (M9942, Sigma-Aldrich), AF647 goat anti-mouse IgG (H + L; A-21235, Thermo Fisher), HRP goat anti-mouse IgG (115-035-003, Jackson), HRP goat anti-rabbit IgG (P0448, Dako), rabbit anti-ADAM22 (PA5-65610, Thermo Fisher), rabbit anti-ADAM23 (C680120, LSBio), rabbit anti-synapsin-1 (ab64581, Abcam), mouse anti-PSD-95 (75-028, NeuroMab), mouse anti-Kv1.1α (75-105, NeuroMab), mouse anti-β-actin (A2228, Sigma-Aldrich). GraphPad Prism_v8, R version 3.5.1 (R Foundation for Statistical Computing, Vienna, Austria) and ggplot2 (Wickham, 2009) were used for statistical analyses and data presentation. Relevant animal procedures were carried out with UK Home Approval under licence P996B4A4E. The study was approved by the Research Ethics Committee (REC16/YH/0013) and all participants gave written consent.

### Data availability

The data that support the findings of this study are available from the corresponding author, upon reasonable request.

## Results

### LGI1 antibodies target multiple epitopes in serum and CSF of patients

From 31 patients (28/31 with limbic encephalitis, including 12/31 with FBDS, 1/31 FBDS only, 1/31 Morvan’s syndrome, 1/31 neuromyotonia/pain), median serum autoantibody levels to membrane-tethered full-length LGI1 were 80-fold higher than CSF (Fig. 1B), consistent with peripheral antibody generation. Across all individuals, serum and CSF showed similar levels of LRR and EPTP reactivities, with the exception of 2/11 CSFs that showed exclusive LRR or EPTP binding (Fig. 1B). Overall, the serum LRR- and EPTP-domain antibody levels strongly correlated with full-length LGI1-antibody levels (*r *=* *0.88, *P < *0.0001; Fig. 1C). There were no correlations between EPTP: LRR antibody ratios and clinical features (syndrome, tumour status, outcomes and relapses, data not shown), and longitudinal EPTP: LRR antibody ratios remained largely stable within 15 individuals treated with varied regimes (Fig. 1D). Therefore, despite multiple immunotherapies and long follow-up durations, binding to both domains was typical in serum, most CSFs, and across recognized LGI1-antibody-associated syndromes.

### Monoclonal LGI1 antibodies are directed against LRR or EPTP domains of LGI1

**Figure 2:**
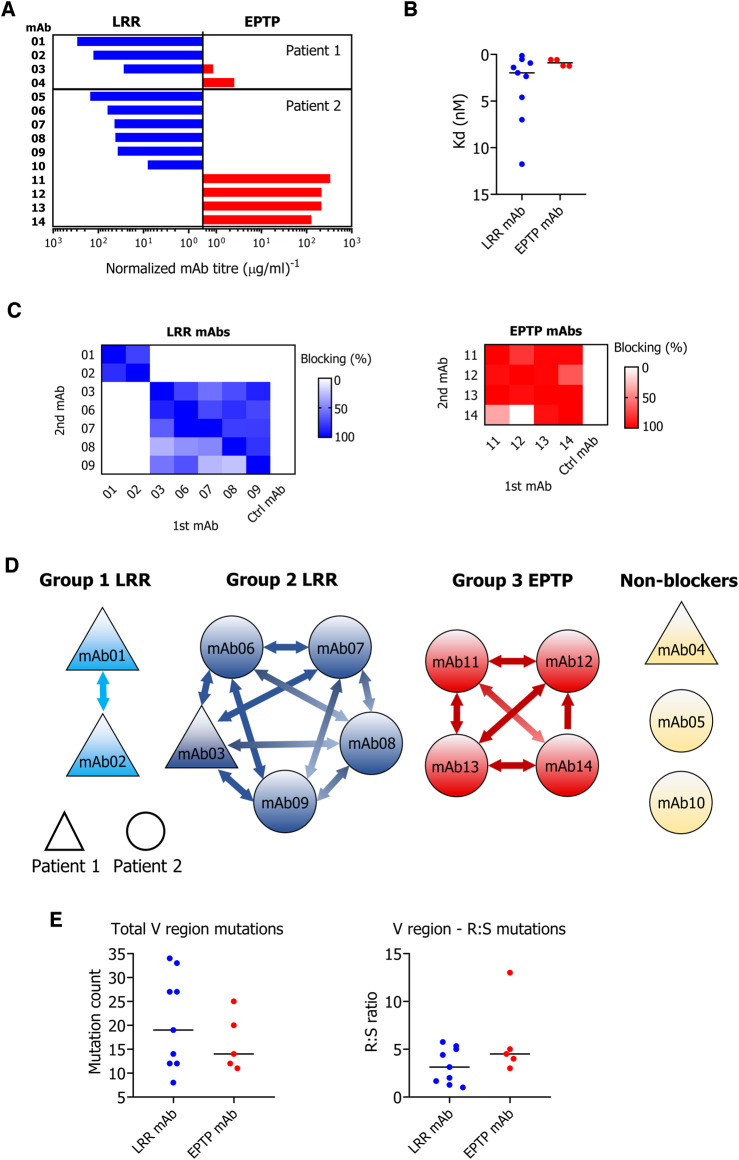
**Epitope specificities and mutation frequencies of patient-derived LGI1 mAbs.** (**A**) Unlike serum, 14 LGI1 mAbs generated from two patients were specific for either LRR or EPTP domains. (**B**) Median relative binding strengths (K_d_), measured by quantification of IgG binding to a live cell-based assay expressing full-length LGI1, were comparable between LRR- and EPTP-specific mAbs. (**C**) Heat maps of two-way blocking experiments for both LRR- and EPTP-binding mAbs. (**D**) From **C**, LRR-specific mAbs clustered into two groups (1 and 2, blue), with cross-competition across mAbs derived from the two patients. EPTP-specific mAbs cross-competed with each other (Group 3, red), except mAb12 did not block mAb14. Three mAbs showed no blocking (non-blockers, yellow). (**E**) Sequences were aligned in IgBLAST against the IMGT reference database. Total and ratios of replacement:substitution (R:S) V region mutations were similar between EPTP- and LRR-binding mAbs.

To examine binding to these domains in greater detail, LGI1-reactive mAbs were generated using fluorescent foci methodology from the PBMCs of two patients (full-length LGI1 IgG serum end-point dilutions: 1:5120 and 1:640, with limbic encephalitis and limbic encephalitis plus FBDS, respectively) (Supplementary Fig. 1A). Immunoglobulin variable region genes were cloned from single B cells, and all 14 recombinant antibodies bound full-length LGI1 in live cell-based assays. Nine of these mAbs bound the LRR and five the EPTP domain, and one showed limited cross-reactivity (Fig. 2A). By analogy to serum, end-point titres of the mAbs against specific domains correlated with their respective end-point titres against full-length LGI1 (Supplementary Fig. 1B). No mAbs bound to full-length CASPR2, ADAM22 or ADAM23 transfected HEK293T cells (Supplementary Fig. 1C), confirming LGI1-specificity.

Binding strengths (K_d_) of mAbs to full-length LGI1 were overall comparable between LRR and EPTP-reactive mAbs (Fig. 2B and Supplementary Fig. 1D). However, two LRR-specific mAbs (mAb05 and mAb10) and one EPTP-specific mAb (mAb04) showed considerably lower maximum binding (B_max_) compared to the other mAbs (Supplementary Fig. 1D). All three lacked self-blocking capacity in cross-competition experiments.

To determine within-domain overlap of epitopes, cross-competition experiments were performed on membrane-tethered full-length LGI1. Among seven LRR-specific mAbs, two of these cross-competed (both from Patient 1), as did another five (four from Patient 2 and one from Patient 1; Fig. 2C). The four EPTP-specific mAbs from Patient 2 cross-competed, with the exception of mAb12, which did not block mAb14 (one-way blocking only; Fig. 2C). Hence, three cross-blocking groups could be identified, suggesting at least two distinct epitopes within the LRR domain, and one dominant EPTP domain epitope (Fig. 2D).

### Peripherally-derived monoclonal LGI1 antibodies are genetically heterogeneous

To explore the nature and diversity of the mAb-specific sequences, their mutation frequencies, germline gene origins, complementarity determining region 3 (CDR3) lengths and sequences were interrogated. All mAbs showed evidence of somatic hypermutation. The total number of mutations across the variable region and the ratio of replacement:silent mutations were comparable for EPTP- and LRR-specific mAbs (Fig. 2E). Furthermore, these 14 mAbs exhibited diverse combinations of variable and joining gene segment usage in both the heavy and paired light chain sequences (Supplementary Fig. 2). Interestingly, however, two LRR-reactive mAbs—one from each patient—expressed the same heavy chain variable and joining allele (mAb01 and mAb10; IGHV3-11*01 and IGHJ4*01; Supplementary Fig. 2A). Also, among the light chain sequences, one combined kappa variable and joining region (IGKV1-5*03 and IGKJ1*01), which natively paired with the mAb10 heavy chain, was shared with an unrelated heavy chain from the same patient (mAb09; IGHV1-69*06 and IGHJ4*02; Supplementary Fig. 2A). Overall, LRR-directed mAbs showed a non-significant trend towards a preference for kappa light chains. Also, CDR3 lengths, a measure correlated with human antibody autoreactivity (Vander Heiden *et al.*, 2017), were not significantly different between the two sets of mAbs (Supplementary Fig. 2B). Moreover, alignments of the cross-competing mAbs revealed no CDR3 sequence homology (Supplementary Fig. 2C).

Taken together, there is notable diversity in the sequences and binding strengths of peripherally-derived LGI1 mAbs. Yet, their overall dichotomy into LRR- versus EPTP-binding antibodies led us to ask whether these two subgroups mediate distinct functional effects.

### LRR-directed antibodies bind and internalize docked LGI1

**Figure 3:**
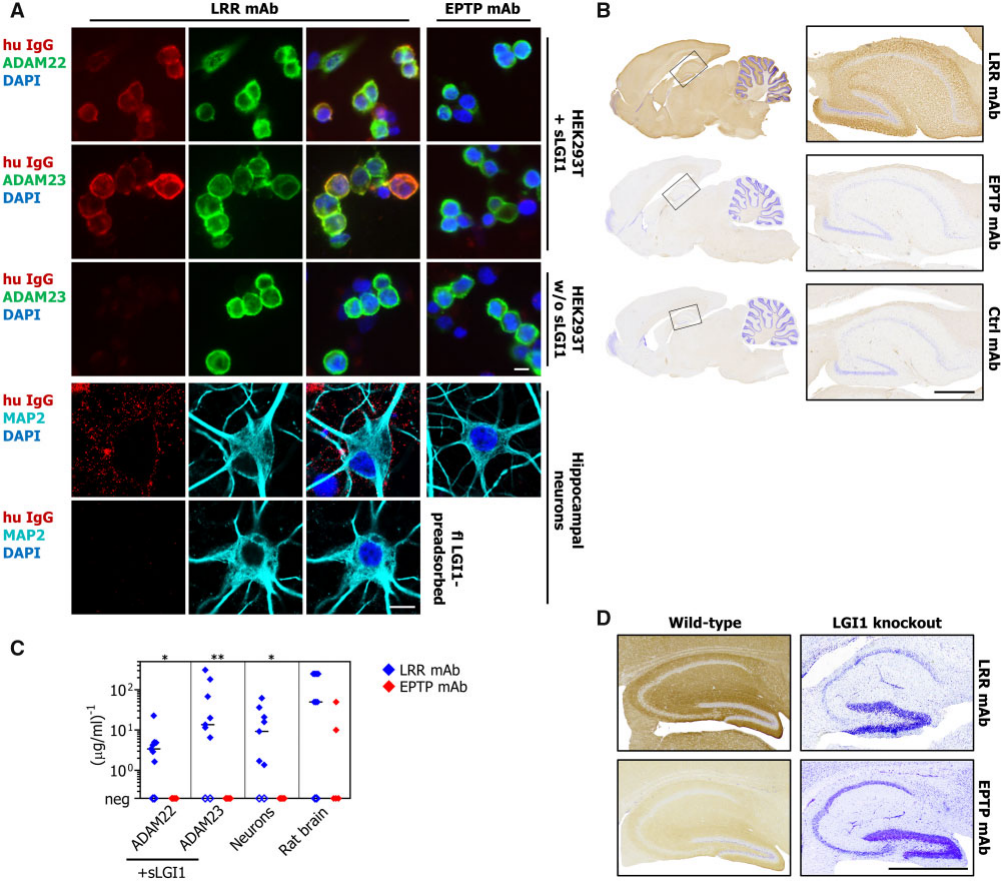
**LRR-, but not EPTP-, specific mAbs bind docked sLGI1.** (**A**) By contrast to EPTP-specific mAbs, 7/9 LRR-specific mAbs recognized LGI1 after it bound the surface ADAM22 or ADAM23 expressed on HEK293T cells (*top two rows*), and to the surface of live hippocampal neurons (*second last row*). They did not directly bind either ADAM22 or ADAM23 without sLGI1 (*third row* and Supplementary Fig. 1C). In hippocampal neuron cultures, binding was abolished after preadsorption of LRR-specific mAbs with HEK293T cells expressing membrane-tethered full-length LGI1 (*bottom row*), but not after preadsorption with untransfected HEK293T cells (not shown). (**B**) Rat brain immunohistochemistry shows an example of sagittal whole brain and hippocampal staining seen in 7/9 LRR-specific mAbs (*top*, mAb09 shown). This was absent for 3/5 EPTP-specific mAbs (*middle*, mAb12 shown) and for a human isotype-control mAb (Ctrl mAb, *bottom*). Similar results were obtained using different fixation methods (paraformaldehyde, formalin, acetone; not shown). All mAbs were tested at ≥5 μg/ml, images at 10 μg/ml. (**C**) Normalized end-point titres of 14 mAbs are shown across all these four detection methods. Using live cell cultures, binding was absent for all EPTP-specific mAbs and 2/9 LRR-directed mAbs (mAb05 and mAb10; open symbols). On rat brain sections, 2/5 EPTP-specific mAbs bound weakly compared to LRR-specific mAbs (e.g. **D**), but with comparable end-point titres (mAb11 binds at 100 ng/ml, mAb13 at 12.5 ng/ml). Representative data from one of two experiments are shown. Medians were compared using Mann-Whitney test *P < 0.05, **P < 0.01. (**D**) The 7/9 LRR-specific mAbs (*top*, mAb08 shown) and 2/5 EPTP-specific mAbs (*bottom*, mAb13 shown), which stained brain tissue from wild-type mice showed no binding to *Lgi1*-knockout mouse sections (at 3 μg/ml). Scale bars = 10 μm in **A**; 1 mm in **B** and **D.**

To assess potentially distinct functionalities, LRR- and EPTP-specific mAb binding was compared after addition of sLGI1 to HEK293T cells expressing ADAM22/23 (Fig. 3A). Using this culture model, 7/9 LRR-reactive mAbs bound to ADAM22/23-docked LGI1, compared to 0/5 EPTP-reactive mAbs. The same 7/9 LRR-reactive mAbs bound to live hippocampal neurons (Fig. 3A). The 2/9 LRR-reactive mAbs that showed no binding to docked LGI1 and live neurons had the lowest B_max_ values in live cell-based assays (Supplementary Fig. 1D; mAb05 and mAb10), consistent with low binding strength. Again, the same seven LRR-reactive mAbs bound to brain sections from wild-type rodents, but not from *Lgi1* knockout mice (Fig. 3B–D). Staining of both the neuronal preparations was abrogated by preadsorption of the mAbs against full-length LGI1-expressing HEK293T cells (shown for hippocampal neurons, Fig. 3A), but not by preadsorption against untransfected HEK293T cells, further verifying their exclusive specificity for LGI1. In contrast to the LRR-directed mAbs, none of the EPTP-specific mAbs bound to live hippocampal cultures (Fig. 3A and C). However, 2/5 EPTP-specific mAbs showed limited binding to rodent brain sections at high concentrations (≥5 µg/ml; Fig. 3C and D), and this binding was abrogated in sections from *Lgi1* knockout mice (Fig. 3D).

**Figure 4:**
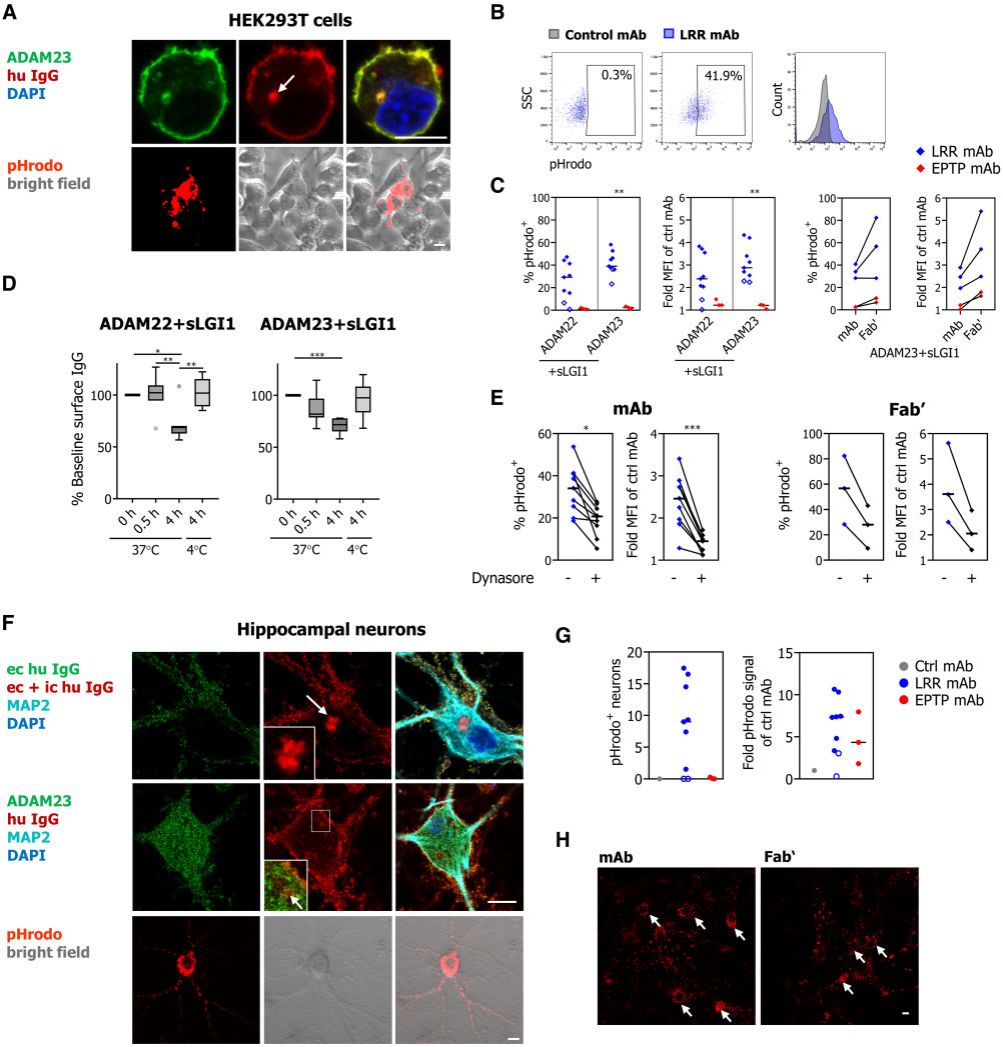
**LRR-specific mAbs internalize LGI1 and its receptors.** (**A**) LRR-specific mAbs caused internalization of sLGI1-ADAM22/23 complexes on HEK293T cells at 37°C after 4 h (*top*: example with ADAM23 staining). *Bottom*: An internalized pHrodo-labelled LRR-specific mAb. Images were similar to ADAM22 (not shown). (**B**) Gating strategy and quantification of pHrodo-labelled mAb uptake (5 μg/ml) by flow cytometry after 4 h incubations at 37°C. (**C**) The percentage of HEK293T cells with pHrodo fluorescence and the fold increase in pHrodo median fluorescent intensity are shown, after incubation with whole mAbs and Fab' fragments (both at 5 μg/ml). Medians of two to four experiments are shown (Mann-Whitney test **P < 0.01). (**D**) Corresponding decrease of surface-bound IgG over time. Graphs summarize the per cent of baseline surface-bound human IgG on sLGI1-ADAM22/23 expressing HEK293T cells after 0.5 and 4 h at 37°C, and after 4 h at 4°C. Data from each time point were compared to their own control values at baseline, and are shown across two experiments using seven LRR-directed mAbs [box plots with median, 25th and 75th percentiles, whiskers indicate 10th and 90th percentiles; repeated measures one-way ANOVA (plus Bonferroni correction); *P < 0.05, **P < 0.01, ***P < 0.001]. (**E**) Quantification of pHrodo fluorescence from conjugated mAbs and Fab′ (5 μg/ml) by flow cytometry with and without dynasore. Medians of two experiments are shown (Mann-Whitney test *P < 0.05, ***P < 0.001). (**F**) LRR-specific mAbs were internalized on live hippocampal neurons at 37°C after 96 h. *Top*: Extracellular (ec), surface human IgG was detected before cell permeabilization (green) and after cell permeabilization [extracellular plus intracellular (ic), red; arrow and *inset*]. *Middle*: ADAM23 staining was observed throughout the neuron (as Ohkawa *et al.*, 2013), and the internalized LRR-directed mAb co-localized with ADAM23 (*inset* and arrow). ADAM22-directed commercial antibodies revealed no binding in these cultures, consistent with 10-fold lower quantities of *ADAM22* mRNA compared to *ADAM23* mRNA (qPCR, data not shown). *Bottom*: Fluorescence and bright field images of hippocampal neurons after 96-h incubation with pHrodo-conjugated LRR-directed mAbs (1 μg/ml). (**G**) Quantification shows the number of pHrodo-positive somatic clusters and relative fluorescence intensities per image [data obtained from nine images (three per well) per condition repeated in two separate cultures]. LRR-directed antibodies formed more somatic pHrodo-positive clusters and fluoresced more intensely than EPTP mAbs, two of which bound rodent brain sections (medians were compared using Mann-Whitney test: not significant, but *P *=* *0.02 for pHrodo-positive somatic clusters if only considering neuron-binding LRR-specific mAbs, filled symbols). Two EPTP antibodies were not tested. (**H**) Representative images of internalized pHrodo-labelled mAb02 (LRR) and its corresponding Fab′ fragments (1 μg/ml) in hippocampal neurons. Arrows indicate somatic clusters. For **C** and **G**, open blue symbols identify the two LRR-specific mAbs that did not bind hippocampal neurons. Scale bars = 10 μm.

Next, ADAM22/23-transfected HEK293T cells loaded with sLGI1 were incubated for 4 h at 37°C with the LRR-directed mAbs. All seven LRR-directed mAbs with detectable binding to sLGI1 (Fig. 3C) internalized and co-localized with intracellular ADAM22/23 (Fig. 4A). After conjugation with the endosomal pH-sensitive dye, pHrodo, internalization of LRR-directed mAbs was observed in ∼30% (sLGI1-ADAM22) or 40% (sLGI1-ADAM23) of HEK293T cells after 4 h (Fig. 4A–C). Uptake of pHrodo-conjugated Fab′ fragments was observed with at least equivalent intensities to whole mAbs (Fig. 4C). Correspondingly, flow cytometry revealed a ∼30% median decrease in residual surface IgG after a 4-h incubation with whole mAbs at 37°C and this effect was inhibited at 4°C (Fig. 4D). Also, dynasore, a molecule that inhibits endocytosis by blocking dynamin activity, markedly reduced mAb and Fab′ fragment uptake (Fig. 4E). By contrast, in this system, EPTP-specific mAbs did not internalize. Taken together, within sLGI1-ADAM22/23 expressing HEK293T cells, LRR-specific mAbs undergo endocytosis in a dynamin- and temperature-dependent process.

In hippocampal neurons, live-cell imaging revealed that LRR-specific mAb internalization could be observed by ∼1 h, but was seen more consistently by 10–12 h and peaked at around 4 days, a time point by which somatic accumulations became evident (Supplementary Video 1 and Supplementary Fig. 3A and B). The IgG exposure did not affect neuron survival (Supplementary Fig. 3C and D). At around 4 days, internalized IgG was detected by immunofluorescence in neurons, and co-localized with diffusely-expressed ADAM23 (Fig. 4F). Analogous to IgG staining observations, from all seven LRR-directed mAbs that bound brain sections and live hippocampal neurons, pHrodo was detected in neuronal somata after 4 days. This uptake was also observed with Fab′ fragments (Fig. 4F–H) but not with an isotype control mAb (Fig. 4G, Supplementary Video 2 and Supplementary Fig. 3A and B). Throughout these experiments, the LRR-specific mAbs that did not bind hippocampal neurons showed the lowest effect sizes (Fig. 4C and G; open circles). Also, and in contrast to the transfected HEK293T cell system, only the 2/5 EPTP-specific mAbs that bound rodent brain sections induced some internalization in hippocampal neurons, but with fewer somatic clusters compared to LRR-specific mAbs (Fig. 4G, Supplementary Videos 3, 4 and Supplementary Fig. 3A and B).

Next, this internalization was confirmed with purified human serum IgG (Thompson *et al.*, 2018). sLGI1-ADAM22/23 co-transfected HEK293T cells and neurons were exposed to purified serum IgG either depleted of or enriched for the IgG4 fraction. By comparison to healthy control IgGs, both the IgG4 enriched and depleted samples showed internalization, under the same conditions used in the mAb experiments (Supplementary Fig. 4A and B). Hence, both LGI1 mAbs and IgG4 LGI1 antibodies from patient sera are taken up by transfected HEK293T cells and hippocampal neurons.

### EPTP-binding antibodies block the interaction of LGI1 with ADAM22/23

**Figure 5:**
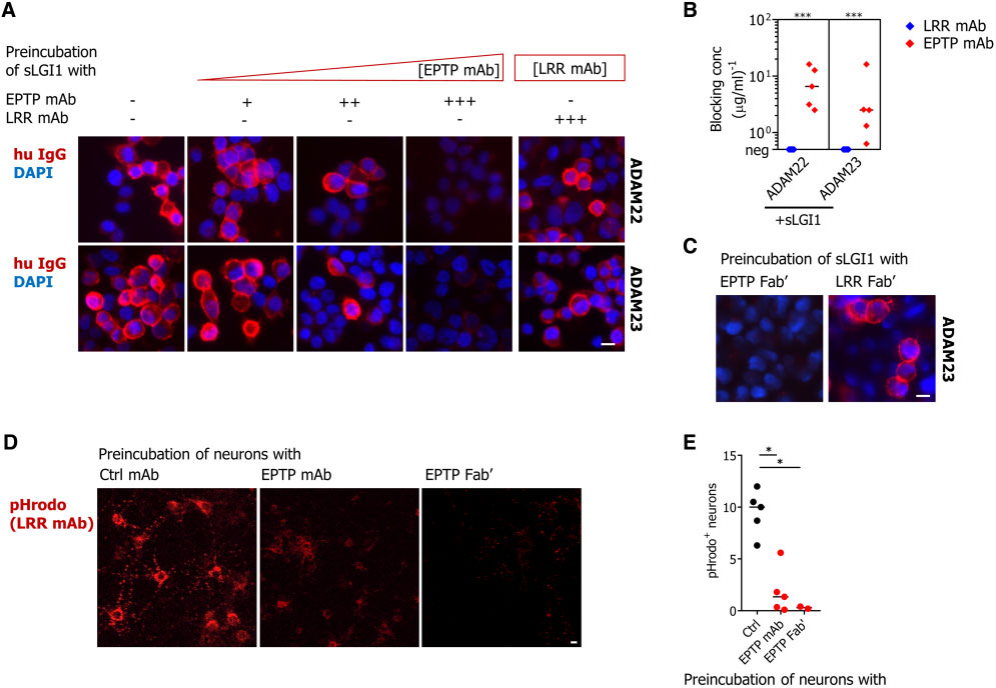
**EPTP-specific mAbs block the interaction of LGI1 with its receptors.** (**A**) Human sLGI1 was preincubated with mAbs, and sLGI1 binding was detected on the surface of ADAM22/23-expressing HEK293T cells with a fluorescently-labelled LRR-specific mAb (mAb02 shown; to assess LRR-specific mAbs within cross-blocking Group 1, a fluorescently-labelled LRR-specific mAb from Group 2 was used). Preincubation with increasing concentrations of all EPTP-specific mAbs (gradient depicted in *left* four panels), but none of the LRR-specific mAbs (*right*-most panel), resulted in a complete loss of fluorescence. (**B**) Individual minimum titres of EPTP-directed mAbs required to achieve complete loss of fluorescence are shown for both ADAM22 and ADAM23-transfected HEK293T cells. No blocking was achieved with LRR-directed mAbs (data from one of three representative experiments are shown; medians were compared using Mann-Whitney test ***P < 0.001). (**C**) Representative images showing EPTP-specific, but not LRR-specific, Fab′ fragments (200 ng/ml) blocked the interaction of sLGI1 with ADAM23 in transfected HEK293T cells. (**D**) Representative images showing that preincubation of hippocampal neurons with an excess of each EPTP-specific mAb individually reduced LGI1 internalization, as visualized with a pHrodo-conjugated LRR-specific mAb (mAb01). Preincubation with an excess of the unconjugated LRR-specific mAb (mAb01) was used as a negative control and displaced all of the observed pHrodo-conjugated mAb01 internalization (not shown). (**E**) Quantification of pHrodo-positive neurons, using all five EPTP-specific mAbs, two EPTP-specific Fab′ fragments and negative controls (anti-A33 mAb, three healthy control IgG preparations and no IgG); representative data obtained from nine images (three per well) per condition repeated in two separate cultures; medians were compared using Kruskal Wallis test (plus Dunn's correction) *P < 0.05. Scale bars = 10 μm.

Given the known docking of LGI1’s EPTP domain to ADAM22/23 (Fukata *et al.*, 2006; Ohkawa *et al.*, 2013; Yamagata *et al.*, 2018), and the observed lack of binding of EPTP-specific mAbs to ADAM22/23-bound sLGI1 and live neuronal cultures, we asked whether EPTP-specific mAbs disrupt the interaction between LGI1 and its receptors. Indeed, preincubation of sLGI1 with EPTP-directed mAbs abrogated the binding of sLGI1 to surface-expressed ADAM22/23 in HEK293T cells, as visualized with LRR-directed mAbs (Fig. 5A). This was observed in a dose-dependent manner across all EPTP-binding mAbs and with EPTP-directed Fab′ fragments (Fig. 5A–C). Finally, to investigate this potential blocking effect within hippocampal neurons, a pHrodo-conjugated LRR-specific mAb was added to the culture to visualize internalization. By comparison to control IgGs or no IgG, preincubation of neurons with an excess of any of the five EPTP-specific mAbs significantly decreased the internalization of LGI1 mediated by an LRR-specific mAb (Fig. 5D and E). Again, as expected, similar blocking effects were observed with EPTP-directed Fab′ fragments on hippocampal neurons (Fig. 5D and E). These findings are consistent with a functional block of endogenous LGI1 binding to the surface of hippocampal neurons mediated by EPTP-specific mAbs.

### LRR- and EPTP-directed LGI1 monoclonal antibodies show pathogenic potential *in vivo*

**Figure 6:**
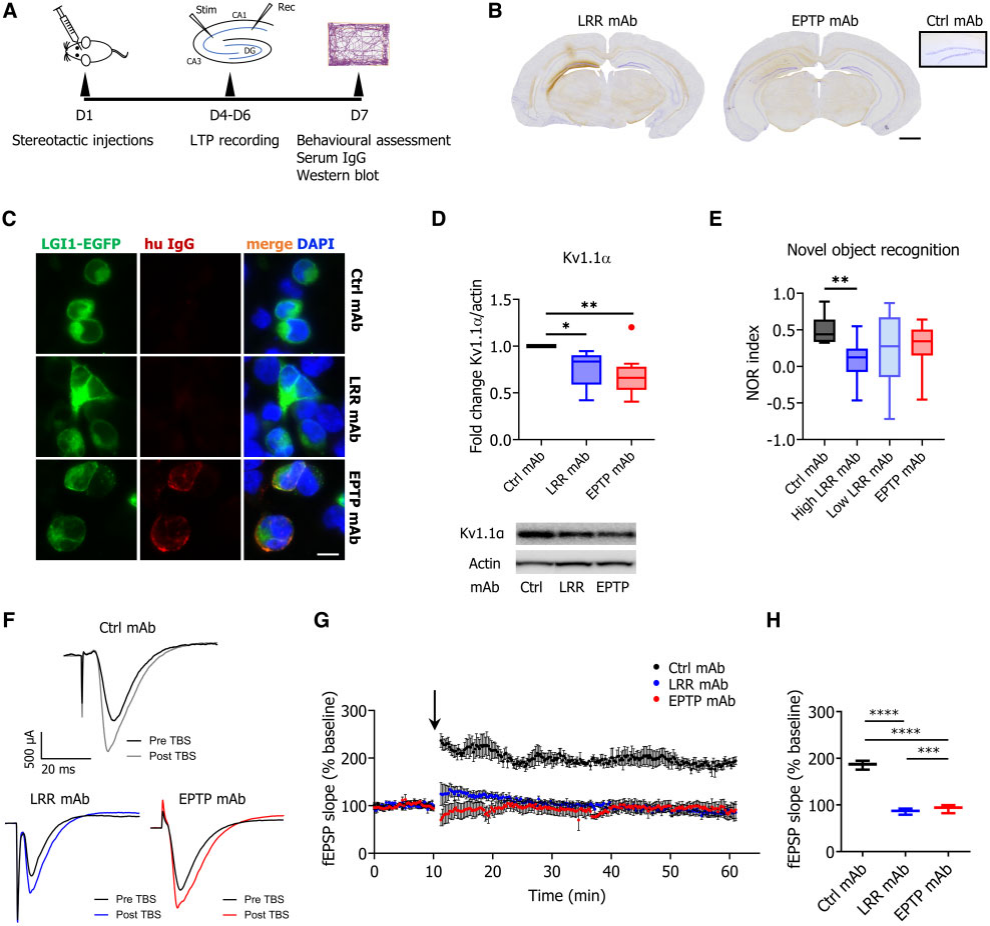
**Single LGI1 mAbs are sufficient to cause pathogenicity *in vivo*.** (**A**) Timeline for stereotactic injections, electrophysiology studies and behavioural assessments. (**B**) Representative staining of human IgG on coronal sections of animals injected unilaterally with LGI1 mAbs to demonstrate the distribution in the hippocampus. Both mAb specificities were absent from the uninjected, contralateral hippocampus, akin to control mAb injections (*inset*). While LRR mAbs were strongly retained in the injected hippocampus, EPTP mAbs showed only limited binding to this tissue. (**C**) Cell-based assays expressing membrane-tethered full-length LGI1 showed the presence of EPTP mAb (all animals tested, mAb13 shown), but not any of the LRR mAb (all animals tested, mAb02 shown) in the serum of injected animals. (**D**) Protein quantification and representative western blots showing a downregulation of K_v_1.1α in hippocampus enriched solubilized brain lysates after injection with LRR- and EPTP-specific mAbs [LRR mAbs tested: mAb02 (*n *=* *6), mAb06 (*n *=* *2), mAb08 (*n *=* *2); EPTP mAbs tested: mAb11 (*n *=* *2), mAb12 (*n *=* *2), mAb13 (*n *=* *6); Ctrl mAb (*n *=* *6)]. Data were normalized to the control mAb band on each blot to correct for inter-experimental variability. (**E**) Novel object recognition testing showed impaired memory in animals injected with either mAb specificity (LRR mAbs tested: mAb02, mAb06, mAb08; EPTP mAbs tested: mAb11, mAb12, mAb13; Ctrl mAb; *n *=* *8 animals per mAb). The effect was significant only for LRR mAbs with high relative binding strengths (mAb02 and mAb06). (**F**) Representative traces from recordings of fEPSPs in hippocampal area CA1 following stimulation of Schaffer collaterals pre and post theta-burst stimulation (TBS). (**G**) Average traces showing LTP following TBS (arrow). Both mAb specificities prevented induction of LTP (control mAb: *n *=* *6; mAb02 and mAb13: *n *=* *5 animals per group). (**H**) Median fEPSP slope 50–60 min after TBS. All data shown as box plots with median, 25th and 75th percentiles, whiskers indicate 10th and 90th percentiles; Kruskal-Wallis test (plus Dunn's correction) *P < 0.05, **P < 0.01, ***P < 0.001, ****P < 0.0001. Scale bars = 1 mm in **B** and 10 μm in **C.**

To assess *in vivo* measures of pathogenicity, six LGI1 mAbs were individually injected under stereotactic guidance into the CA3 region of the hippocampus of mice. Synaptic LTP, a proposed cellular correlate of learning and memory (Nicoll, 2017), was assessed from brain slices at Days 4–6. This was followed by behavioural tests plus serum sampling and western blotting at Day 7 (Fig. 6A).

As expected, injected LRR-specific mAbs bound strongly within the hippocampus. In contrast, EPTP-specific mAbs showed only weak staining of the hippocampus (Fig. 6B), similar to our results from rodent brain sections (Fig. 3B–D). Consistent with this, after the intracerebral injection only the EPTP-specific mAbs were detectable in mouse serum (Fig. 6C), suggesting the LRR mAbs were more efficiently adsorbed by the brain.

At Day 7 after injection, K_v_1.1α protein levels in hippocampus enriched brain tissue were decreased after injection of either LRR- or EPTP-specific mAbs (Fig. 6D). By contrast, no changes were observed in ADAM23, PSD-95 and synapsin-1 protein levels (Supplementary Fig. 5A). Overall, the LRR mAbs impaired novel object recognition, an effect that was accounted for by the two with higher relative binding strengths (mAb02 and mAb06; Fig. 6E), and not seen after injection of the EPTP-directed mAbs. For all mAbs, no changes were observed in measures of locomotor activity (distance travelled) and anxiety (open field test; Supplementary Fig. 5B and C), and no seizures were witnessed. Also, LTP was assessed by recording field potentials at CA3–CA1 synapses in acute hippocampal slices. Strikingly, there was a complete failure of LTP induction following theta-burst stimulation of the Schaffer collateral pathway in mice injected with an LRR- or EPTP-specific mAb, while robust LTP was observed with a control mAb (Fig. 6F–H). Neither LGI1 mAb induced alterations in paired-pulse facilitation, suggesting that presynaptic release probability was unchanged (Supplementary Fig. 5D).

## Discussion

The generation of several mAbs against LGI1 has dissected the pathogenic potential of individual components of humoral immunity at both the molecular and systems levels. Our observations highlight the concept that highly diverse mAbs exist in patients. Even within populations directed against single epitopes, these may have widely varying binding characteristics and pathogenic potentials both *in vitro* and *in vivo*.

Overall, two major groups of antibodies emerged. The LRR-directed antibodies show varied epitope specificities, binding strengths and mutation frequencies. After targetting ADAM22/23-docked LGI1, robustly binding antibodies mediated internalization of the antigen-receptor complex with widely varying magnitudes. This effect was not dependent on the bivalency of IgG; it was retained with Fab fragments alone and with IgG4-enriched serum fractions. Hence, our data suggest this effect can be mediated *in vivo* by IgG4 molecules that Fab-arm exchange (Kolfschoten *et al.*, 2007). This potentially monovalent mechanism is an area for further investigation, and contrasts with the absence of internalization reported with Fab fragments derived from NMDAR-IgGs (Hughes *et al.*, 2010). In terms of definitive *in vivo* pathogenic effects, intrahippocampal administration of the LRR antibodies with higher relative binding strength led to impairment of recognition memory in mice, and resulted in a substantial abrogation of LTP induction at CA3–CA1 synapses. These observations may reflect the likely hippocampus-mediated amnesia seen in patients with LGI1 antibodies. They are consistent with previous work on the effects of polyclonal LGI1 antibodies on CA3–CA1 synapses (Miller *et al*., 2017;Petit-Pedrol *et al.*, 2018), whereas elsewhere in the hippocampus, LGI1 antibodies appear to mediate additional changes in basal synaptic strength and cellular excitability (Petit-Pedrol *et al.*, 2018; Kornau *et al.*, 2020).

In striking contrast, the EPTP-specific antibodies did not bind to receptor-docked LGI1 in dissociated live neuronal cultures or in HEK293T cells. Therefore, in these two preparations the majority of available LGI1 already appears to be docked, via its EPTP domain. However, we did observe weak binding of some EPTP-specific antibodies to brain sections, as did other independent investigators using CSF-derived LGI1 mAbs (Kornau *et al.*, 2020). Indeed, these tissue-binding EPTP-directed mAbs were taken up by neurons and perhaps they are binding to exposed EPTP domains of multimerized LGI1 within the synaptic cleft or extracellular network. However, their dominant effect appears to be inhibition of secreted LGI1 binding to membrane-bound and soluble ADAM22/23 (Kornau *et al.*, 2020). Their limited binding to brain tissue was also observed after *in vivo* injection, and from their consistent detection in the periphery after intracerebral injections. The restricted observed CNS adsorption may account for the relatively minor effect EPTP-directed antibodies had on behavioural measures, despite their striking effect on LTP.

While we observed discrete domain-specific mechanisms, the frequent presence of both specificities in the serum and CSF of patients evokes questions about their potential synergistic contributions to disease pathology. Indeed, the neutralization of pathogens is often enhanced by several orders of magnitude with polyclonal antibody preparations versus mAbs alone (He *et al.*, 2015; Gilchuk *et al*., 2020). Therefore, the presence of both LRR- and EPTP-specific antibodies may cumulatively enhance disruption of LGI1 function. Also, alternative molecular mechanisms likely operate. For example, the LGI1-ADAM22/23 complex has been shown to form heterotetrameric trans-synaptic assemblies where the LRR domain of one LGI1-ADAM complex interacts with the EPTP domain of the other (Yamagata *et al.*, 2018). The LRR-specific mAbs, which bound pre-docked LGI1, may therefore play a role in disruption of proposed higher order assemblies of the LGI1-ADAM22/23 complex (Yamagata *et al.*, 2018). This, and currently undiscovered additional molecular mechanisms, could further explain the clear memory impairment by the LRR-specific mAbs with higher binding strength seen in our *in vivo* model. Future experiments may also focus on the lower-frequency complement fixing IgG1-mAbs (Thompson *et al.*, 2018), which were not studied with our IgG4 mAbs, and relative downstream pre- versus postsynaptic effects of the antibodies (Ohkawa *et al.*, 2013; Petit-Pedrol *et al.*, 2018). Other general limitations of the current work, and similar mAb-based studies, include the use of supra-physiological concentrations of mAbs (Jurek *et al.*, 2019; Kornau *et al.*, 2020), and the absence of a phenotype which fully mimics the patients: indeed, seizures are probably the most distinctive hallmark of patients with LGI1 antibodies and were not observed in our model.

For clinical diagnostic purposes, our data suggest that assays which use pre-docked LGI1 may fail to detect many EPTP-binding antibodies. From our sample, no sera but 2/11 of CSFs showed exclusive binding to a single domain. So, these biologically intriguing observations provide insights into the highest sensitivity diagnostics.

Our data also have immunological implications. First, the diversity of genetic sequences encoding the LRR- or EPTP-targeting mAbs both between and within patients suggest a generalized breakdown of B cell tolerance, rather than expansion of a single or few rogue autoreactive B cell clone(s). Second, the accumulation of multiple somatic hypermutations is consistent with traditional models of germinal centre reactions (Makuch *et al.*, 2018; Wilson *et al.*, 2018), as opposed to the unmutated, yet functionally active NMDAR antibodies (Kreye *et al.*, 2016; Wenke *et al.*, 2019). Such germinal centre reactions are likely initiated by specific T cells that interact with the strongly associated HLA-DRB1*07:01 molecule (Kim *et al.*, 2017; van Sonderen *et al.*, 2017; Binks *et al.*, 2018; Mueller *et al.*, 2018).

Overall, in this study, focused molecular hypotheses have been accurately dissected via the generation of patient-derived mAbs. The findings have highlighted intriguing aspects of the immunology and neuroscience, which inform multiple mechanisms underlying this highly amnesic and epileptogenic disease. While several mechanisms remain to be explored, these findings provide insights into the potential of mAbs to inform many aspects of the underlying disease biology which could not easily be highlighted by the study of polyclonal sera and CSFs.

## Supplementary Material

awaa104_Supplementary_Data

## Abbreviations

EPTP =: epitempin repeat domain
FBDS =: faciobrachial dystonic seizure
IgG =: immunoglobulin G
LRR =: leucine-rich repeat
LTP =: long-term potentiation
mAb =: monoclonal antibody
sLGI1 =: soluble LGI1
