# Supplementary material for: Distinctive binding properties of human monoclonal LGI1 autoantibodies determine pathogenic mechanisms
Source: Brain. 2020 May 21;143(6):1731–45. doi: 10.1093/brain/awaa104 (PMC7296845; doi:10.1093/brain/awaa104)
Supplement: awaa104_Supplementary_Data [file awaa104_supplementary_data.zip › awaa104_Supplementary_Data/OP-BRAI200105_EditorCorr_CmtAttachmentsFolder_awaa104 Supplementary_material final.pdf]

Supplementary Figure 1. Monoclonal antibody generation and their binding characteristics to full length LGI1 in live cell-based assays. (A) Depiction of fluorescent foci method used to generate LGI1 antigen-specific monoclonal antibodies (mAbs) from patient B cells (Clargo *et al.*, 2014). (B) mAb titres to full-length (fl) LGI1 (range 5-800 ng/ml), and LRR or EPTP domains, showed a significant correlation (Spearman's r = 0.65, p = 0.01). (C) Representative images showing ADAM22 in transfected HEK293T cells without the addition of sLGI1 is not recognized by LRR- and EPTP-specific mAbs. Scale bar 10 μm. (D) Quantification of mAbs binding to live HEK293T cells expressing full-length membrane-tethered LGI1, with mAb titrations. Graphs show absolute fluorescence intensities. After subtraction of background fluorescence intensity (Ctrl mAb), a one site nonlinear regression model was used to calculate maximum binding (Bmax*) and relative binding strength (Kd).

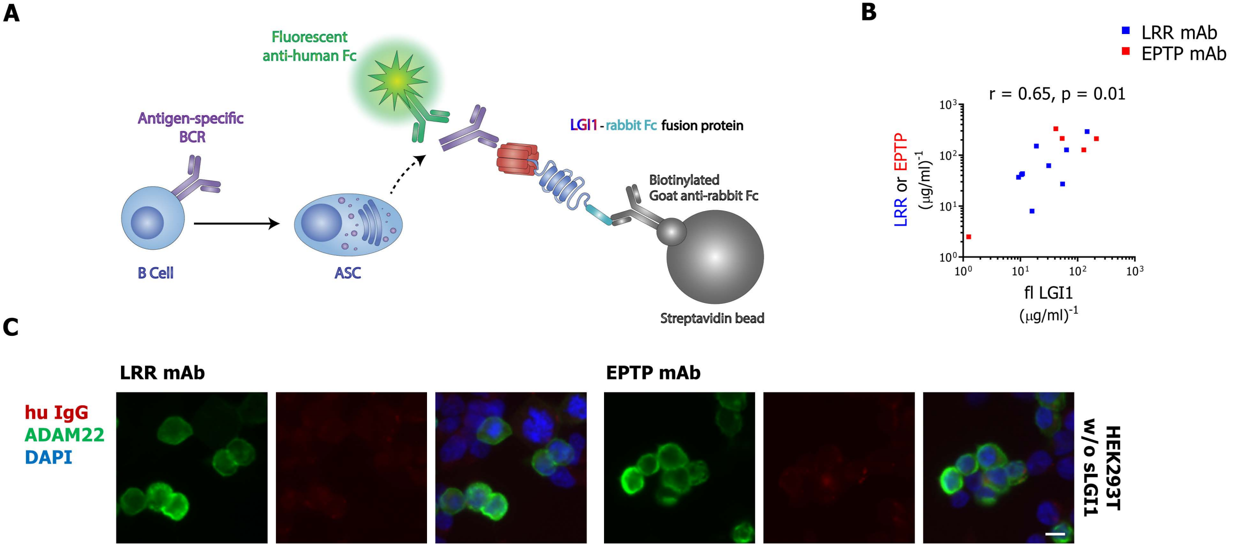

D

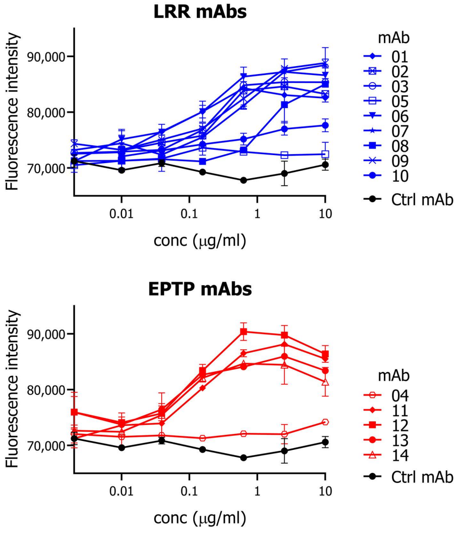

mAb	Bmax*	Kd (nM)
01	13,066	0.52
02	16,098	2.35
03	16,939	1.97
04	nd	nd
05	2,858	0.13
06	16,404	0.92
07	22,206	7.00
08	17,810	11.76
09	18,891	4.59
10	4,658	1.40
11	19,900	1.21
12	20,352	1.23
13	14,819	0.56
14	15,296	0.57

Supplementary Figure 2. LGI1-specific monoclonal antibody sequence characteristics. (A) Sequences were aligned in IgBLAST against the IMGT reference database. Monoclonal antibodies showed very limited overlaps in IGHV and IGHJ genes. (B) CDR3 region amino acid (AA) lengths across domain-specific mAbs in heavy (IGH), kappa (IGκ) and lambda (IGλ) chains (Fisher's exact and Mann-Whitney tests not significant). (C) Amino acid sequences of heavy chain CDR3.

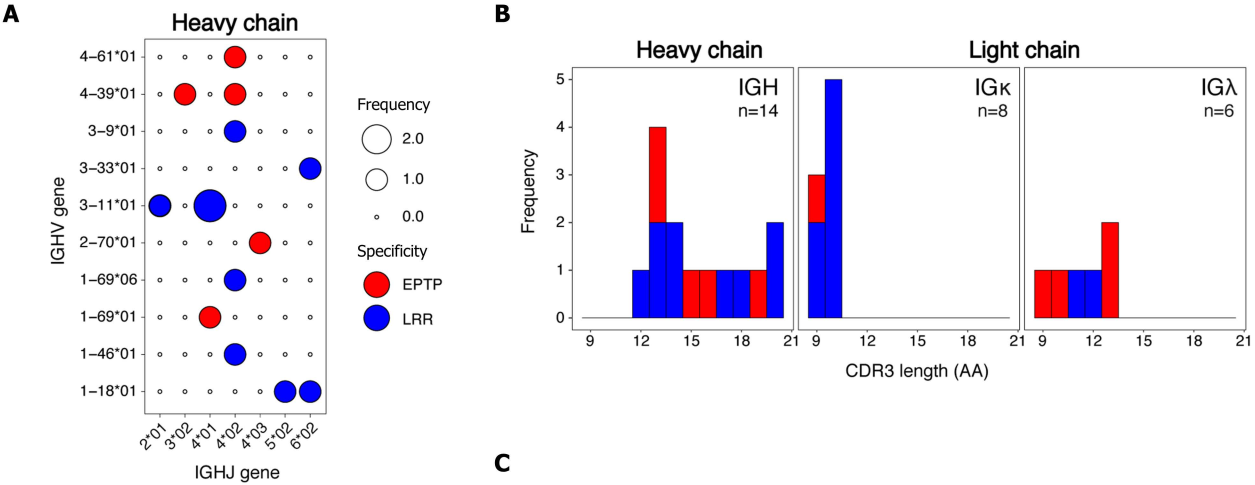

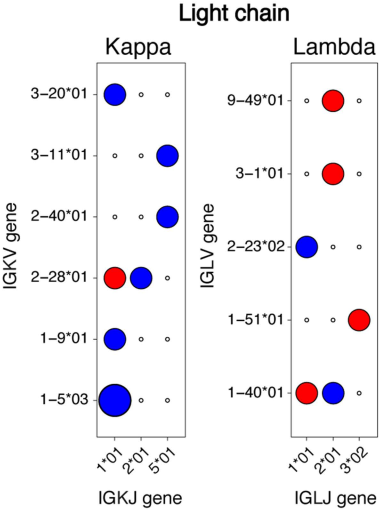

C

mAb	Specificity	Heavy chain CDR3
01	LRR	CARDRGDGAYYFDVW
02	LRR	CARDLEGYSFGSFDQW
03	LRR	CARWASPRIGPMLRGGLDPW
04	EPTP	CARPSVGIVVPFDIW
05	LRR	CARVRGVLYFFDYW
06	LRR	CARDRFPFRPEWVGGMDVW
07	LRR	CAKDLFPYCSDITCALNGFGSW
08	LRR	CAREGIRTSGSVGHYYHGMDVW
09	LRR	CARFYYDSGGDPFDYW
10	LRR	CARVTEAVVGPIDFW
11	EPTP	CARVTIAVATTAYFDYW
12	EPTP	CVRHLEVRYFDWSSDTHFDYW
13	EPTP	CARGGWYCSSGSCYFDLW
14	EPTP	CARGKWALAGAYDYW

Supplementary Figure 3. Computational analysis of internalized pHrodo conjugated monoclonal antibodies (mAbs) in hippocampal neurons and neuronal viability after four days. (A) Quantification of pHrodo fluorescence intensity in live cell imaging over time (Ctrl mAb, 1 LRR- (mAb02) and 2 EPTP-specific mAbs (mAb12 and mAb13; 1 µg/ml; see also Suppl Videos 1-4). The grey area depicts the 95% confidence interval for the intensity curve. (B) Representative images at 96 hours showing the absence (Ctrl mAb) or presence (high frequency: LRR mAb; low frequency: EPTP mAb) of pHrodo positive neurons, indicated by the accumulation of somatic clusters (arrows). (C) Representative images and (D) quantification of thresholded MAP2 staining on hippocampal neurons after 4 days incubation with LGI1 mAbs (LRR: mAb01 and EPTP: mAb13; 5 µg/ml). Data were obtained from 9 images (3 per well), and are shown as box plots with median, 25th and 75th percentiles, whiskers indicate 10th and 90th percentiles. Scale bars = 10 µm.

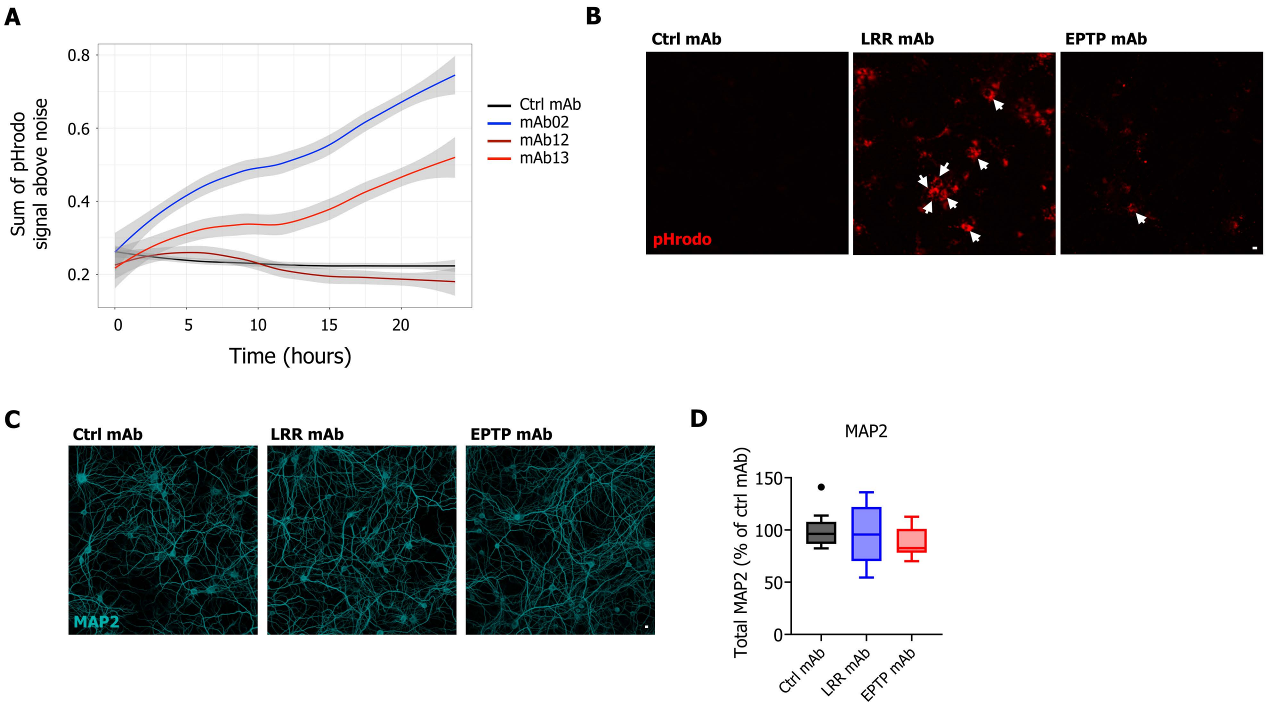
A
Sum of pHrodo signal above noise
0.8
0.6
0.4
0.2
0
5
10
15
20
Time (hours)
Ctrl mAb
mAb02
mAb12
mAb13
B
Ctrl mAb
LRR mAb
EPTP mAb
pHrodo
C
Ctrl mAb
LRR mAb
EPTP mAb
MAP2
D
MAP2
Total MAP2 (% of ctrl mAb)
150
100
50
0
Ctrl mAb
LRR mAb
EPTP mAb

Supplementary Figure 4. LGI1-specific patient IgG4 is internalized. (A) Gating strategy and flow cytometry quantification of pHrodo-conjugated purified serum IgG depleted of the IgG4 fraction (IgG1-3) and enriched for IgG4. In both fractions, after 4 hours at 37°C, patient IgG internalized sLGI1 bound to its receptors, ADAM22 and ADAM23, in transfected HEK293T cells compared to healthy control (HC) IgG fractions. Pooled data from 4 patients and 2 HC on ADAM22 and ADAM23 transfected HEK293T cells are shown (Mann-Whitney test * $p < 0.05$, ** $p < 0.01$). (B) In hippocampal neurons, the IgG4 fraction from patient 2, which contains the vast majority of LGI1-specific autoantibodies, showed higher pHrodo fluorescence intensity compared to their IgG1-3 fraction and HC IgG (all at 125 µg/ml) after 4 hours at 37°C. The difference became more apparent at later time points, as shown by computational analysis of live cell imaging as in Suppl Fig 3. Scale bars = 10 µm.

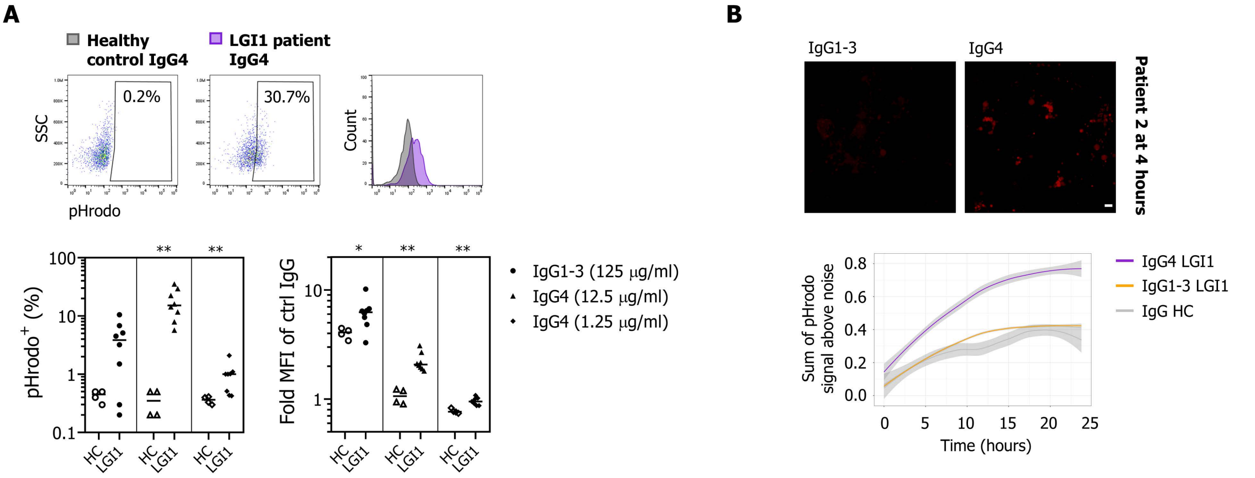

A
Healthy control IgG4
LGI1 patient IgG4
0.2%
30.7%
SSC
pHrodo
Count
pHrodo+ (%)
Fold MFI of ctrl IgG
HC
LGI1
IgG1-3 (125 μg/ml)
IgG4 (12.5 μg/ml)
IgG4 (1.25 μg/ml)
B
IgG1-3
IgG4
Patient 2 at 4 hours
Sum of pHrodo signal above noise
Time (hours)
IgG4 LGI1
IgG1-3 LGI1
IgG HC

Supplementary Figure 5. LGI1 monoclonal antibodies (mAbs) have no impact on ADAM23, PSD-95 and synapsin-1 protein levels, locomotor activity, anxiety or neurotransmitter release probability. (A) Representative Western blots and quantification showing unchanged ADAM23, PSD-95 and synapsin-1 protein levels after injection of LGI1 mAbs (LRR mAbs tested: mAb02 [n=5], mAb06 [n=2], mAb08 [n=2]; EPTP mAbs tested: mAb11 [n=2], mAb12 [n=2], mAb13 [n=5]; Ctrl mAb [n=5]). (B) Representative track plots for assessment of locomotor activity. (C) No significant differences in distance travelled (locomotion), or open field (anxiety) tests were observed (LRR mAbs tested: mAb02, mAb06, mAb08; EPTP mAbs tested: mAb11, mAb12, mAb13; Ctrl mAb; n=8 animals per mAb). (D) In day 4-6 electrophysiology studies, paired pulse ratio, a measure that reflects neurotransmitter release probability, was unchanged (control mAb: n=6; mAb02 and mAb13: n=5 animals per group). All data shown as box plots with median, 25th and 75th percentiles, whiskers indicate 10th and 90th percentiles.

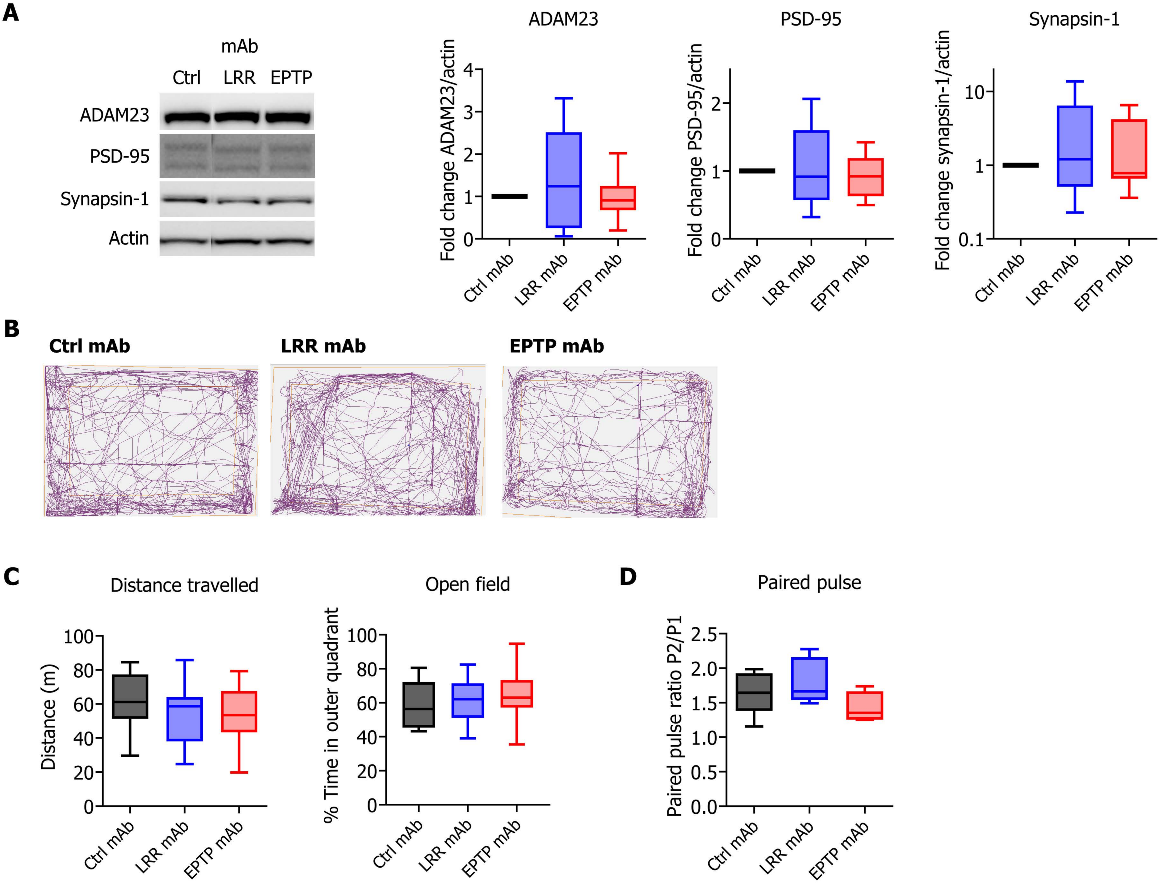
A
mAb
Ctrl LRR EPTP
ADAM23
PSD-95
Synapsin-1
Actin
ADAM23
Fold change ADAM23/actin
PSD-95
Fold change PSD-95/actin
Synapsin-1
Fold change synapsin-1/actin
Ctrl mAb
LRR mAb
EPTP mAb
B
Ctrl mAb
LRR mAb
EPTP mAb
C
Distance travelled
Distance (m)
Open field
% Time in outer quadrant
D
Paired pulse
Paired pulse ratio P2/P1

Supplementary Video Legends

Supplementary Video 1. Time-lapse live cell imaging reveals LRR-specific mAbs are taken up by hippocampal neurons. Representative video shows pHrodo-conjugated LRR-specific mAb02 (1 μg/ml) is taken up by cultured hippocampal neurons. Quantification of pHrodo fluorescence over time is shown in Suppl Fig 3A. Top right shows time in hours.

Supplementary Video 2. Time-lapse live cell imaging reveals control mAbs are not taken up by hippocampal neurons. Representative video shows pHrodo-conjugated control mAb targeting A33 (1 μg/ml) is not taken up by cultured hippocampal neurons. Quantification of pHrodo fluorescence over time is shown in Suppl Fig 3A. Top right shows time in hours.

Supplementary Video 3. Time-lapse live cell imaging reveals non-binding EPTP-specific mAbs are not taken up by hippocampal neurons. Representative video shows pHrodo-conjugated EPTP-specific mAb12 (1 μg/ml) is not taken up by cultured hippocampal neurons, and comparable to the control mAb (Suppl Video 2). Quantification of pHrodo fluorescence over time is shown in Suppl Fig 3A. Top right shows time in hours.

Supplementary Video 4. Time-lapse live cell imaging reveals low level binding EPTP-specific mAbs are taken up by hippocampal neurons. Representative video shows pHrodo-conjugated EPTP-specific mAb13 (1 μg/ml) is taken up by cultured hippocampal neurons, but at considerably lower intensity compared to LRR-specific mAbs (Suppl Video 1). Quantification of pHrodo fluorescence over time is shown in Suppl Fig 3A. Top right shows time in hours.
