# Supplementary figures and images for: Distinctive binding properties of human monoclonal LGI1 autoantibodies determine pathogenic mechanisms
Source: Brain. 2020 May 21;143(6):1731–45. doi: 10.1093/brain/awaa104 (PMC7296845; doi:10.1093/brain/awaa104)

Fig 6D

Kv1.1α

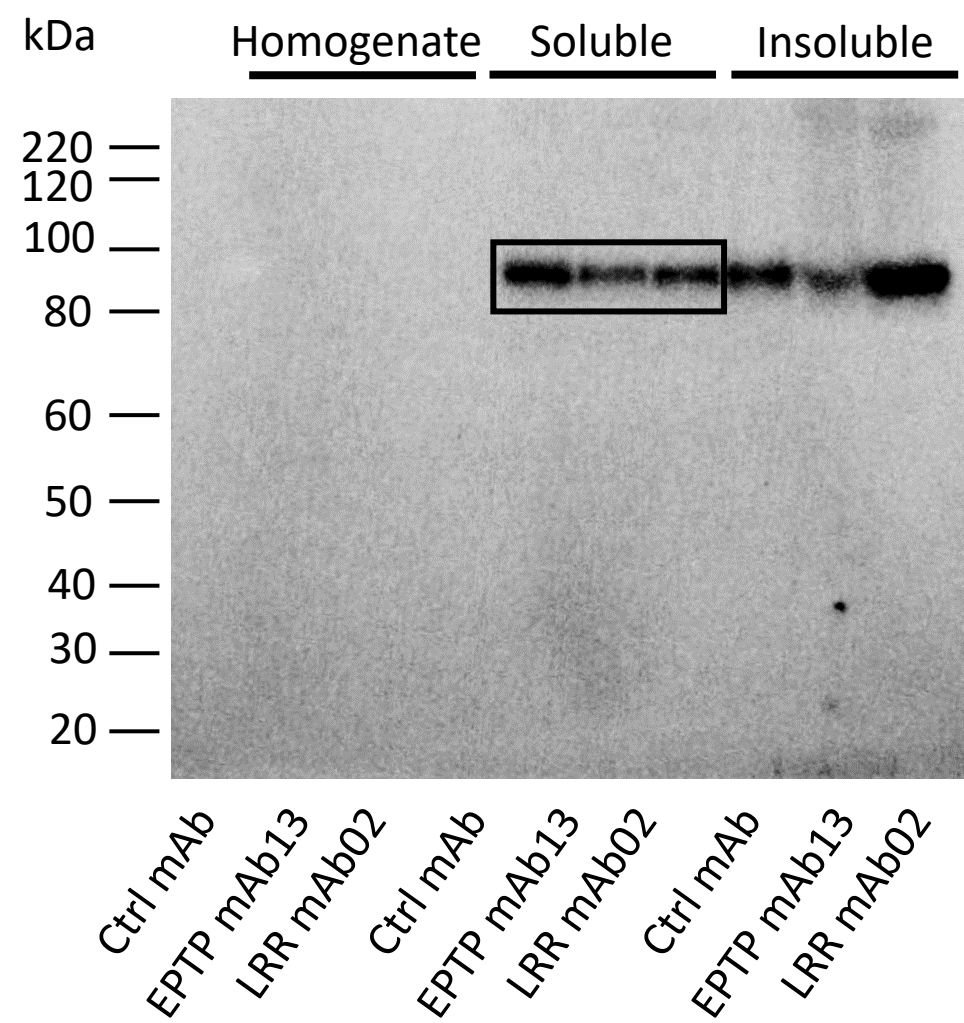

Actin

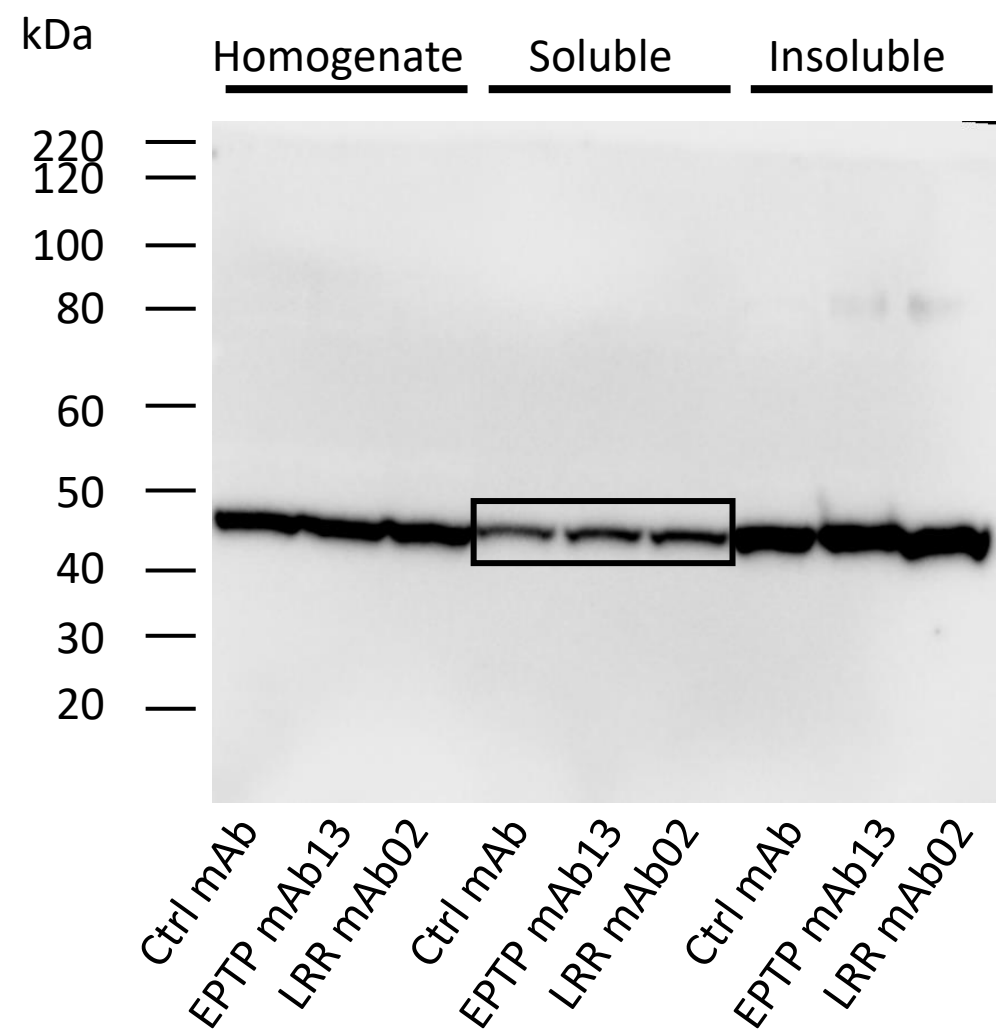

Suppl Fig 5A

ADAM23

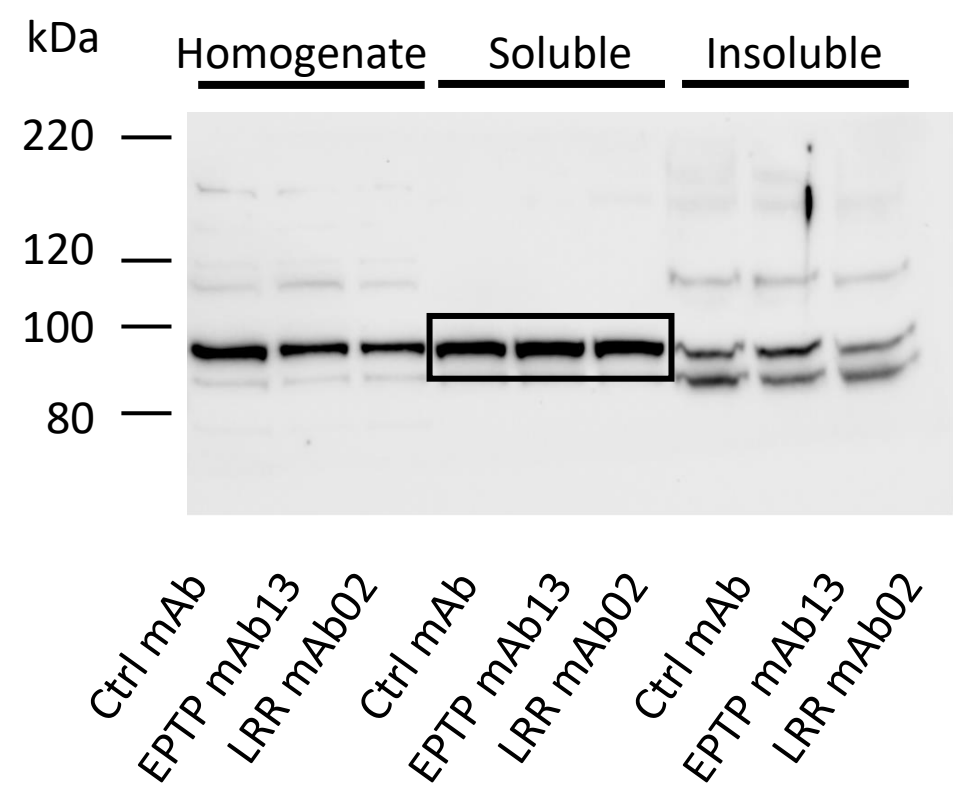

PSD-95

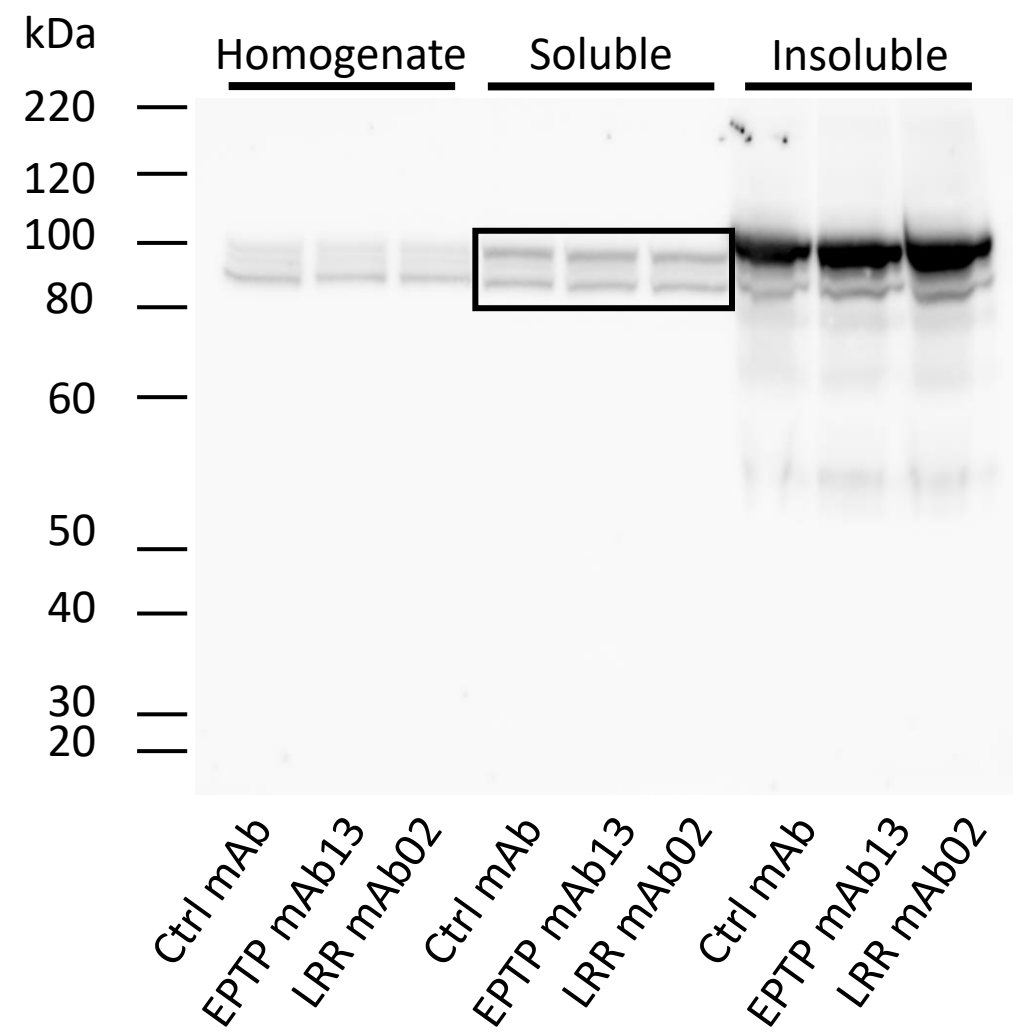

Synapsin-1

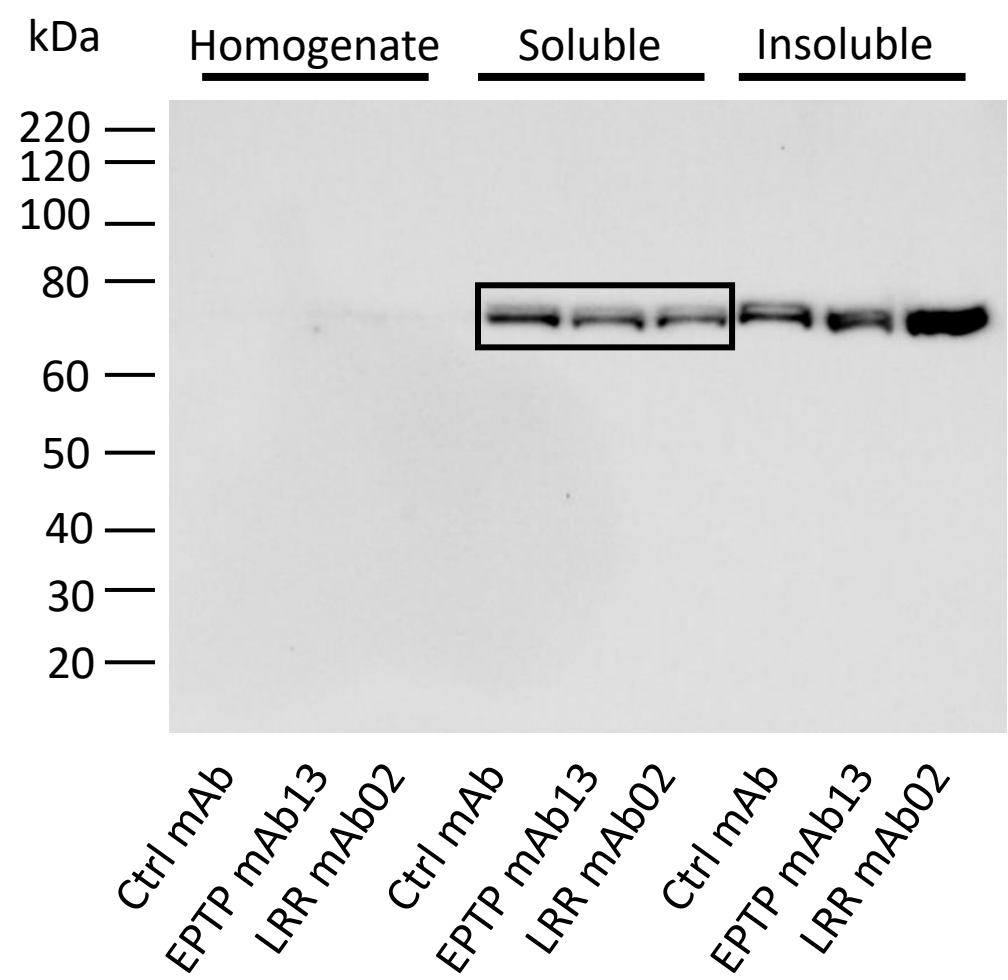

Supplement: awaa104_Supplementary_Data [file awaa104_supplementary_data.zip › awaa104_Supplementary_Data/OP-BRAI200105_EditorCorr_CmtAttachmentsFolder_Suppl uncropped Western blots final.pdf]
